# LSD1 is essential for oocyte meiotic progression by regulating CDC25B expression in mice

**DOI:** 10.1038/ncomms10116

**Published:** 2015-12-02

**Authors:** Jeesun Kim, Anup Kumar Singh, Yoko Takata, Kevin Lin, Jianjun Shen, Yue Lu, Marc A. Kerenyi, Stuart H. Orkin, Taiping Chen

**Affiliations:** 1 Department of Epigenetics and Molecular Carcinogenesis, The University of Texas MD Anderson Cancer Center, Science Park, 1808 Park Road 1C, Smithville, Texas 78957, USA; 2 Center for Cancer Epigenetics, The University of Texas MD Anderson Cancer Center, Science Park, 1808 Park Road 1C, Smithville, Texas 78957, USA; 3 Division of Hematology/Oncology, Boston Children's Hospital and Dana-Farber Cancer Institute, Harvard Stem Cell Institute, Harvard Medical School, 450 Brookline Avenue, Boston, Massachusetts 02115, USA; 4 Howard Hughes Medical Institute, Harvard Medical School, 450 Brookline Avenue, Boston, Massachusetts 02115, USA

## Abstract

Mammalian oocytes are arrested at prophase I until puberty when hormonal signals induce the resumption of meiosis I and progression to meiosis II. Meiotic progression is controlled by CDK1 activity and is accompanied by dynamic epigenetic changes. Although the signalling pathways regulating CDK1 activity are well defined, the functional significance of epigenetic changes remains largely unknown. Here we show that LSD1, a lysine demethylase, regulates histone H3 lysine 4 di-methylation (H3K4me2) in mouse oocytes and is essential for meiotic progression. Conditional deletion of *Lsd1* in growing oocytes results in precocious resumption of meiosis and spindle and chromosomal abnormalities. Consequently, most *Lsd1*-null oocytes fail to complete meiosis I and undergo apoptosis. Mechanistically, upregulation of CDC25B, a phosphatase that activates CDK1, is responsible for precocious meiotic resumption and also contributes to subsequent spindle and chromosomal defects. Our findings uncover a functional link between LSD1 and the major signalling pathway governing meiotic progression.

## Abstract (web summary)

At puberty, hormones stimulate mammalian oocytes to resume meiosis, which is linked to dynamic epigenetic changes, but what controls such changes are unknown. Here, Kim *et al*. identify LSD1, a lysine demethylase, as regulating histone methylation at meiotic progression and also female fertility.

## Graphical abstract

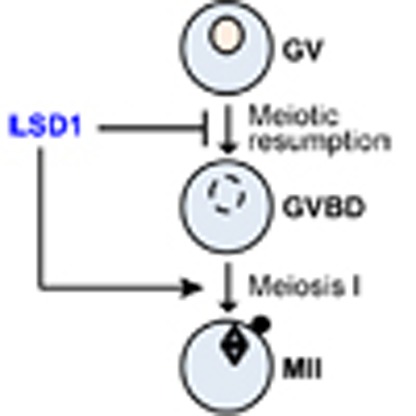

Meiosis is a specialized cell cycle that occurs in sexually reproducing organisms. This process, which consists of two successive cell divisions, meiosis I and meiosis II, after a single round of DNA replication, halves the chromosome compliment in gametes. In most mammals, meiosis is initiated in female germ cells during fetal life, but the process is arrested at prophase of meiosis I (prophase I) around the time of birth. Even though oocytes grow significantly in size during folliculogenesis, prophase I arrest remains in effect until puberty when luteinizing hormone (LH) induces resumption of meiosis^1^,^2^. Oocytes arrested at prophase I have an intact nuclear envelope known as the germinal vesicle (GV). GV breakdown (GVBD) marks resumption of meiosis and, after proceeding through metaphase I, anaphase I and telophase I, the oocyte extrudes the first polar body, which marks the completion of meiosis I. Then, the oocyte directly enters meiosis II and becomes arrested for a second time at metaphase II (MII) before being released at ovulation. Resumption and completion of meiosis II occur following fertilization.

A major regulator of meiotic progression is the maturation-promoting factor (MPF), a complex consisting of the cyclin-dependent kinase 1 (CDK1, also known as CDC2) and a regulatory subunit cyclin B1 (refs 3, 4). CDK1 is inactivated when it is phosphorylated on Thr14 and Tyr15. In mouse oocytes, the WEE2 (formerly WEE1B) kinase and the CDC25B phosphatase mediate CDK1 phosphorylation and dephosphorylation, respectively^4^. In the follicular environment, oocytes maintain high levels of cyclic adenosine monophosphate (cAMP), which activates protein kinase A (PKA) that in turn phosphorylates WEE2 and CDC25B, resulting in WEE2 activation and CDC25B cytoplasmic retention^5^,^6^,^7^. The combined effect maintains low levels of CDK1 activity required for sustaining prophase I arrest. The preovulatory LH surge leads to a significant decrease in oocyte cAMP, which triggers meiotic resumption by alleviating phosphorylation of WEE2 and CDC25B. Genetic studies have demonstrated that CDC25B is essential for CDK1 activation and the resumption of meiosis in mice^8^. During meiosis, CDC25B levels show marked fluctuations^9^,^10^, suggesting that proper regulation of CDC25B expression is important for meiotic progression. How CDC25B expression is regulated during meiosis is not well understood.

Meiotic progression is accompanied by epigenetic changes. For example, histone H3 and H4 are globally deacetylated during meiotic maturation, and H3 Lys4 (H3K4) and Lys9 (H3K9) methylation exhibit dynamic changes during early stages of meiosis^11^,^12^,^13^. Conceptually, epigenetic events may ensure the appropriate expression of genes involved in meiosis, contribute to chromosome integrity and chromosome dynamics associated with meiotic progression, and prepare the genome for gene expression in the embryo^14^. However, little is known about the functional significance of epigenetic changes, the key epigenetic factors regulating these changes, and the crosstalk between epigenetic regulation and the signalling pathways involved in oocyte meiotic progression.

Lysine-specific demethylase 1 (LSD1), also known as lysine (K) demethylase 1A (KDM1A), was the first lysine-specific demethylase identified^15^. It is an evolutionarily conserved enzyme, specific for mono- and di-methyl marks on H3K4 and/or H3K9 (H3K4me1/2 and/or H3K9me1/2), that plays crucial roles in the germ line of multiple organisms^16^. In *Drosophila*, the LSD1 ortholog Su(var)3-3 (also known as dLSD1), a suppressor of heterochromatic silencing, demethylates H3K4 and is essential for fertility in both male and female. Its loss leads to a complete absence of oocytes in female flies and severe defects in spermatogenesis in male flies^17^,^18^. In *Caenorhabditis elegans*, disruption of the *Lsd1* ortholog *spr-5* leads to progressive male and female sterility over many generations, resulting from failure to erase H3K4me2 in primordial germ cells^19^. In mammals, there are two LSD1 family members, LSD1 and LSD2 (also known as KDM1B). They primarily demethylate H3K4 (refs 15, 20), although LSD1 has also been shown to demethylate H3K9 when associated with androgen receptor^21^. LSD2 is specifically expressed in growing oocytes in mice and plays an essential role in the establishment of maternal DNA methylation imprints^20^. LSD1, which is widely expressed during development and in somatic tissues, is essential for mouse embryogenesis^22^,^23^, and its role in the germ line has not been explored.

Here we present evidence that LSD1 controls global H3K4me2 levels in oocytes and is essential for fertility of female mice. Conditional deletion of *Lsd1* in growing oocytes substantially compromises the capacity to sustain prophase I arrest, largely due to upregulation of CDC25B. *Lsd1*-null oocytes also exhibit increased DNA damage, derepression of retrotransposons, spindle and chromosomal defects, and aneuploidy, with the majority undergoing apoptosis before the completion of meiosis I. Our results thus demonstrate that LSD1 plays an essential role in the regulation of CDC25B expression and chromatin structure in mammalian oocytes.

## Results

### LSD1 controls global H3K4me2 in developing oocytes

**Figure 1:**
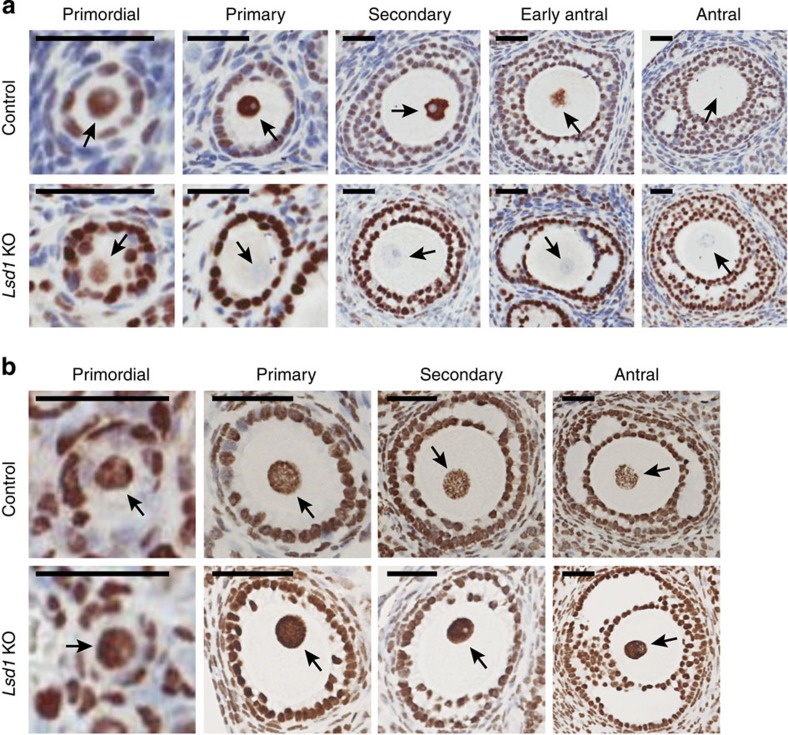
LSD1 regulates global H3K4me2 in growing oocytes. (**a**) Immunohistochemical (IHC) analysis showing the levels of LSD1 in oocytes of primordial, primary, secondary and antral follicles. Ovaries from 6-week-old control and *Lsd1* KO mice were stained with anti-LSD1 and then counterstained with haematoxylin. The nuclei of oocytes are indicated by arrows. (**b**) IHC analysis of H3K4me2 in developing oocytes of control and *Lsd1* KO mice. Scale bars, 25 μm.

To explore whether LSD1 may play a role in the female germ line, we first examined its expression pattern during oocyte development. Immunohistochemical (IHC) analysis of paraffin-embedded sections of ovaries revealed that LSD1 was highly expressed in the nuclei of oocytes of all preantral (primordial, primary, and secondary) follicles (Fig. 1a). However, on the formation of follicular antra, when oocytes acquire meiotic competence (that is, the ability to resume meiosis), LSD1 levels in oocytes drastically decreased and often became undetectable shortly afterwards (Fig. 1a). Although it is unclear how LSD1 levels are regulated in oocytes, the data suggested a potential role for LSD1 during meiotic progression.

To assess the role of LSD1 in the female germ line, we decided to delete *Lsd1* in oocytes. Because LSD1 deficiency results in early embryonic lethality^22^,^23^, we used the Cre-*lox*P technology to disrupt *Lsd1* by crossing mice bearing the *Lsd1*^*fl*^ conditional allele^24^ with *Zp3-Cre* transgenic mice, which expresses the Cre recombinase exclusively in growing oocytes^25^ (Supplementary Fig. 1a). *Lsd1*^*fl/fl*^*/Zp3-Cre*^*+*^ female mice were used as the experimental group, and for simplicity, they will be referred to as *Lsd1* knockout (KO) mice hereafter. Mice of the other genotypes (*Lsd1*^*fl/+*^*/Zp3-Cre*^*−*^*, Lsd1*^*fl/fl*^*/Zp3-Cre*^*−*^*, Lsd1*^*fl/+*^*/Zp3-Cre*^*+*^) produced from the breeding scheme exhibited no overt phenotypes, and *Lsd1*^*fl/+*^*/Zp3-Cre*^*−*^ female mice were used as the control group. Genotypes were determined by PCR (Supplementary Fig. 1b). IHC analysis of *Lsd1* KO ovarian sections revealed that LSD1 was absent in all growing and fully grown oocytes (although it was detected in some non-growing oocytes of primordial follicles, consistent with the timing of *Zp3*-Cre expression), whereas LSD1 levels were not altered in granulosa cells (Fig. 1a). Western blot analysis confirmed the absence of LSD1 in GV oocytes (see Fig. 3a) and no alterations in LSD1 levels in granulosa cells, as well as in various somatic tissues, from *Lsd1* KO mice (Supplementary Fig. 1c). Thus, *Zp3*-Cre-mediated deletion was oocyte specific and the deletion efficiency was apparently 100%, as reported previously^25^.

LSD1 has been shown to erase mono- and di-methyl marks on H3K4 and H3K9 (refs 15, 21). To assess the impact of LSD1 depletion on these histone marks in oocytes, we performed IHC analysis using ovarian sections. As shown in Fig. 1b, *Lsd1* KO oocytes had substantially elevated H3K4me2 levels, as compared with control oocytes, at all developmental stages, with the exception of non-growing oocytes of primordial follicles (when *Lsd1* deletion had not occurred). In contrast, H3K4me1, H3K9me1, and H3K9me2 showed comparable levels in *Lsd1* KO and control oocytes (Supplementary Fig. 2a). Thus, LSD1 mainly demethylates H3K4me2 in oocytes. The observation that oocytes at the antral follicle stage, which normally has little LSD1 (Fig. 1a), also exhibited H3K4me2 elevation with *Lsd1* deletion suggested that the H3K4 hypermethylation state that occurred in preantral oocytes persisted to later stages.

Various histone marks often influence each other and show coordinated changes. We therefore examined several other histone marks that have been well characterized in oocytes^26^. Consistent with the notion that H3K4me2 correlates with open chromatin, the levels of acetylation at H3K9 (H3K9ac) and H3K27 (H3K27ac) were elevated in *Lsd1* KO oocytes (Supplementary Fig. 2b). In contrast, phosphorylation at H3 Ser10 (H3S10ph) and H3 Ser28 (H3S28ph), which are present and absent in GV oocytes, respectively^26^, exhibited no alterations in the absence of LSD1 (Supplementary Fig. 2c).

### LSD1 depletion results in precocious meiotic resumption

*Lsd1* KO females were capable of mating with wild-type males, as evidenced by the presence of vaginal plugs. However, none of them produced offspring, whereas littermate control mice had normal numbers of pups (Supplementary Fig. 3a), indicating that maternal LSD1 is essential for fertility. To determine the cause(s) of infertility, *Lsd1* KO females were superovulated and mated with wild-type males, and embryos were collected at various time points during preimplantation development. While no embryos were recovered from most *Lsd1* KO mice, small numbers (<3) of zygotes were occasionally observed, which apparently could not develop to the two-cell stage (Supplementary Fig. 3b). We next asked whether LSD1 deficiency affected oocyte development. When primed by pregnant mare's serum gonadotropin (PMSG) at 4–6 weeks of age, *Lsd1* KO and control mice produced comparable numbers of morphologically normal GV oocytes. However, significantly fewer MII oocytes were recovered from superovulated *Lsd1* KO mice, as compared with control mice (Supplementary Fig. 3b). These observations suggested that LSD1 is not essential for oocyte growth, but may be important for meiotic maturation after GVBD.

**Figure 2:**
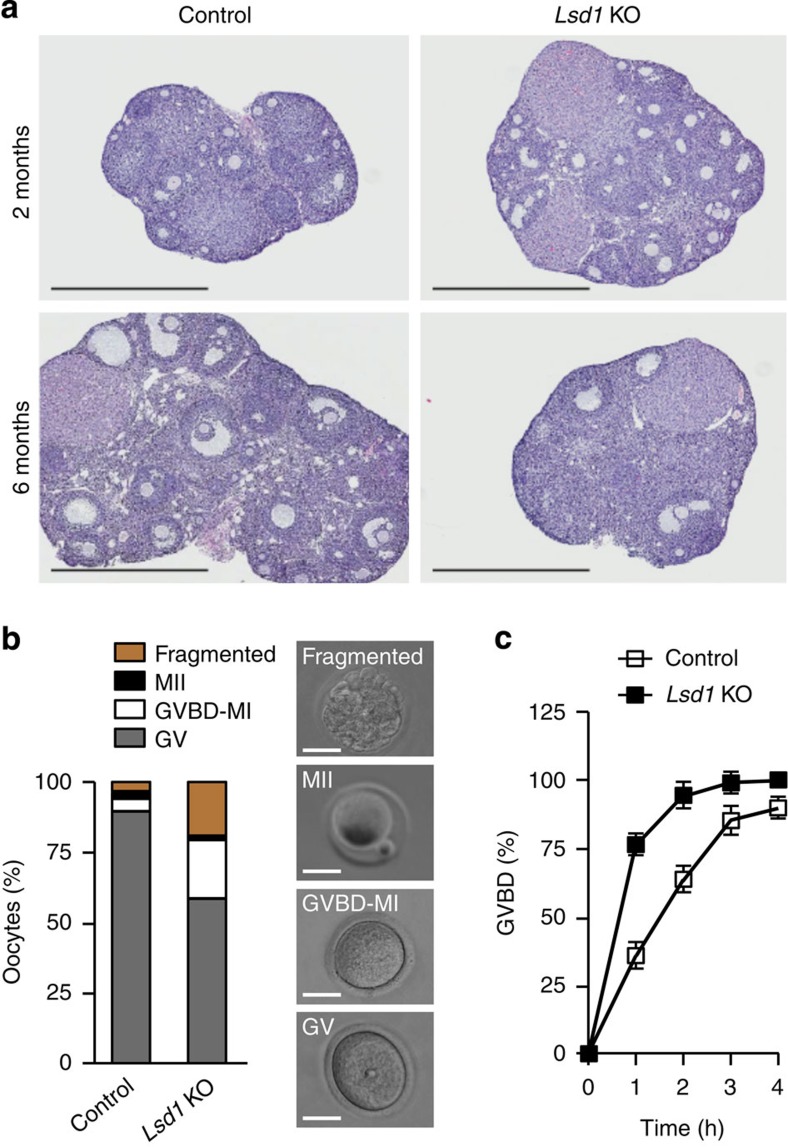
LSD1 depletion in oocytes results in precocious meiotic resumption. (**a**) Haematoxylin and eosin (H&E) staining of ovarian sections. Shown are representative images of ovaries from 2-month- and 6-month-old control and *Lsd1* KO mice. Scale bars, 1 mm. (**b**) Fully grown GV oocytes isolated from ovaries were cultured in the presence of 200 μM IBMX for 20 h and classified as being GV arrested (based on the presence of germinal vesicle; grey bar), GVBD-MI (based on the absence of both germinal vesicle and polar body; white bar), MII (based on the presence of a polar body; black bar) or fragmented (yellow bar). The average proportion of oocytes at each stage from three experiments is plotted as a percentage of the total (left). Examples of GV, GVBD-MI, MII and fragmented oocytes are shown (right). Scale bars, 50 μm. (**c**) Fully grown GV oocytes were collected in M2 medium containing IBMX and, following IBMX washout, the oocytes were cultured in the absence of IBMX and examined hourly to determine GVBD rates. The data at each time point represent the mean±s.e.m. of three experiments.

To investigate how LSD1 loss affects oocyte maturation, we compared the follicular development in control and *Lsd1* KO mice. Ovaries from 1-month-old KO mice exhibited no obvious abnormalities in morphology and histology, with follicles at different stages, as well as corpus lutea (Supplementary Fig. 4). At 2 months of age, KO ovaries were moderately larger than control ovaries, with the presence of more advanced-stage follicles, suggesting that oocyte development was accelerated in the absence of LSD1. Afterwards, control ovaries continued to grow and, by 6 months of age, had reached much larger sizes with many fully grown oocytes. In contrast, *Lsd1* KO ovaries exhibited no obvious changes in size between 2 and 6 months and, by 6 months, ovarian follicles had been largely depleted (Fig. 2a). These observations suggested that LSD1 loss led to accelerated depletion of the oocyte pool, consistent with a premature ovarian aging phenotype.

Prophase I arrest is important for sustaining the oocyte pool^27^,^28^. To determine whether the premature ovarian aging phenotype was due to meiosis defects, we assessed the impact of LSD1 depletion on meiotic progression. Fully grown GV oocytes, when removed from their follicular environment, undergo spontaneous meiotic resumption^29^, which can be reversibly inhibited by cAMP phosphodiesterase inhibitors such as 3-isobutyl-1-methylxanthine (IBMX). Following 20 h of culture in IBMX-containing medium, the vast majority (∼90%) of oocytes isolated from control mice remained arrested at prophase I with intact GV, whereas only ∼60% of *Lsd1* KO oocytes were at prophase I, ∼20% had undergone GVBD and another ∼20% were fragmented (Fig. 2b). We also compared the kinetics of GVBD in the absence of IBMX. GV oocytes collected in IMBX-containing medium were washed extensively before culturing. As shown in Fig. 2c, *Lsd1* KO oocytes underwent GVBD substantially more rapidly, with GVBD rates reaching ∼75% in 1 h (as opposed to ∼30% in control oocytes) and nearly 100% in 2–3 h (as opposed to 60–80% in control oocytes). Taken together, these results indicated that the capacity of oocytes to sustain prophase I arrest was compromised in the absence of LSD1.

### CDC25B upregulation contributes to meiotic defects

**Figure 3:**
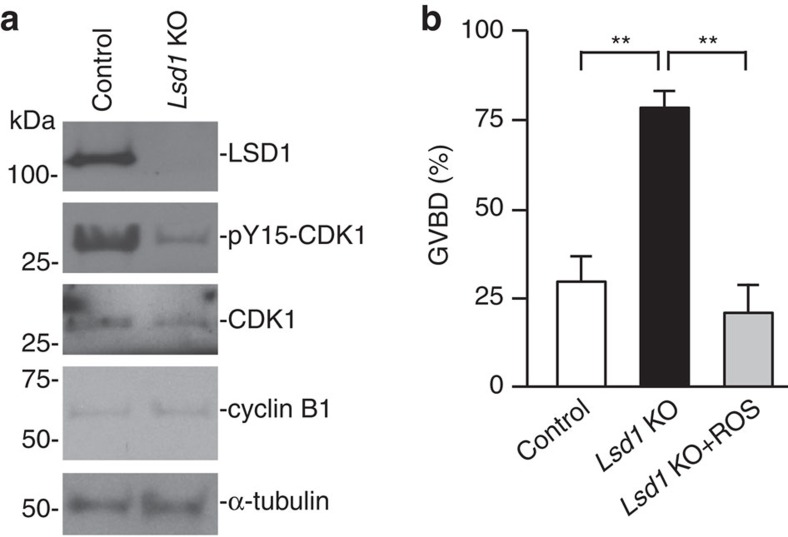
*Lsd1* KO oocytes resume meiosis due to abnormal activation of CDK1. (**a**) Western blot analysis of LSD1, phospho-Tyr15-CDK1 (pY15-CDK1), CDK1 and cyclin B1. α-tubulin serves as a loading control. Each lane contained 100 fully grown GV oocytes. Full blots are provided in Supplementary Fig. 9. (**b**) Fully grown GV oocytes were collected in medium containing IBMX and, following IBMX washout, the oocytes were cultured in the presence or absence of the CDK1 inhibitor roscovitine (ROS, 50 μM) for 1 h to determine GVBD rates. The data represent the mean±s.e.m. of three experiments. Statistical comparisons of values were made using one-way ANOVA. **P<0.01.

Meiotic resumption is controlled by the activity of MPF, consisting of CDK1 and cyclin B1 (refs 3, 4). We therefore asked whether LSD1 loss affected the expression and/or activity of CDK1 and cyclin B1. Western blot analysis revealed that GV oocytes from *Lsd1* KO mice had normal levels of CDK1 and cyclin B1, but significantly reduced phosphorylation of CDK1 at Tyr15, compared to control GV oocytes (Fig. 3a). Because Tyr15 phosphorylation leads to inhibition of CDK1 activity^5^,^30^, our results indicated that MPF was abnormally activated in *Lsd1* KO oocytes. To determine the contribution of CDK1 activation to the observed meiotic phenotype, we tested the effect of roscovitine, a CDK1 inhibitor, on GVBD rates in oocytes cultured in the absence of IBMX. As shown in Fig. 3b, roscovitine prevented the enhanced GVBD rates in *Lsd1* KO oocytes, thus confirming that CDK1 activation was responsible for precocious meiotic resumption caused by LSD1 depletion.

**Figure 4:**
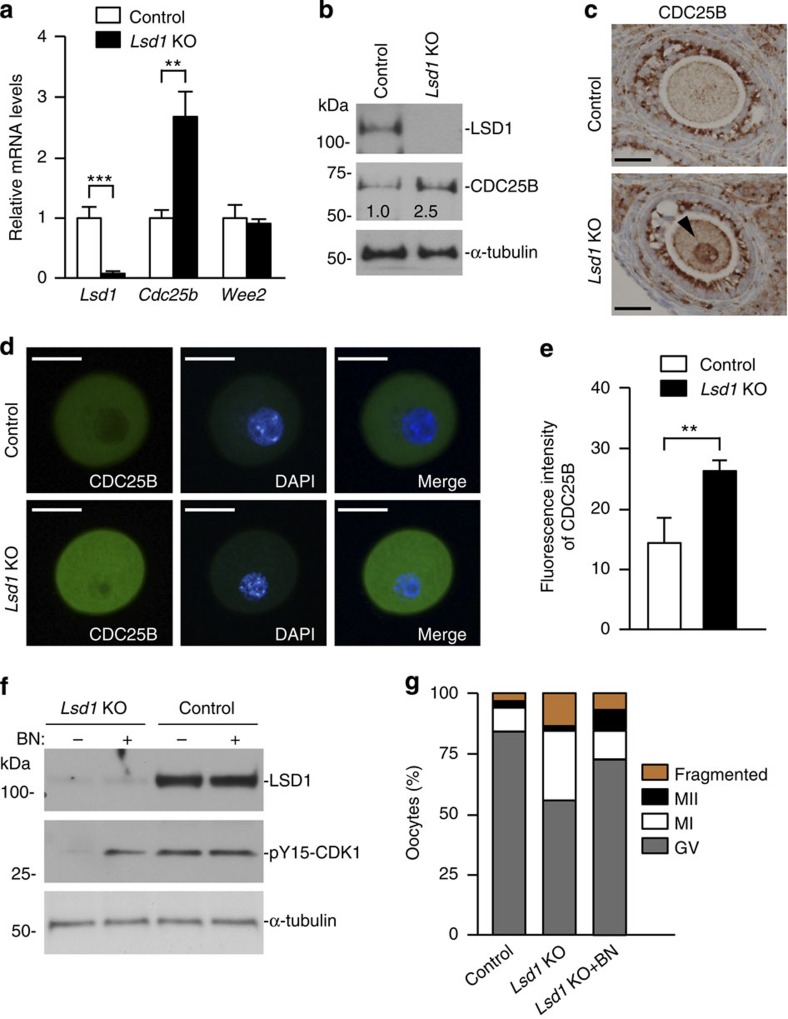
Upregulation of CDC25B contributes to precocious meiotic resumption. (**a**) Quantitative RT–PCR analysis of *Lsd1, Cdc25b* and *Wee2* transcripts in control and *Lsd1* KO GV oocytes. Data are presented as the mean±s.e.m. from three experiments. Statistical comparisons of values were made using multiple *t*-test. **P<0.01; ***P<0.001. (**b**) Western blot analysis of LSD1, CDC25B and α-tubulin (loading control) proteins in control and *Lsd1* KO GV oocytes. Each lane contained 100 GV oocytes. Band intensities were quantified with the ImageJ software and normalized to the α-tubulin signal. Full blots are provided in Supplementary Fig. 10. (**c**) IHC analysis of ovaries from control and *Lsd1* KO mice showing the levels of CDC25B in oocytes of follicles. The arrowhead indicates CDC25B accumulation in the nucleus of an *Lsd1* KO oocyte. Scale bars, 25 μm. (**d**,**e**) Immunofluorescence (IF) analysis of CDC25B in GV oocytes. (**d**) Representative images of control and *Lsd1* KO oocytes stained with anti-CDC25B (green) and DAPI (blue). Scale bars, 40 μm. (**e**) Quantification of fluorescence intensity of CDC25B. 20 control and 28 *Lsd1* KO oocytes were analysed, respectively, and the data are presented as the mean±s.e.m.. Statistical comparisons of values were made using unpaired *t*-test. **P<0.01. (**f**) Fully grown GV oocytes collected from *Lsd1* KO and control mice were cultured for 6 h in IBMX-containing medium with or without the CDC25 phosphatase inhibitor BN82002 (BN), as indicated, and then analysed by immunoblotting with antibodies against LSD1, pY15-CDK1 and α-tubulin (loading control). Each lane contained 100 oocytes. Full blots are provided in Supplementary Fig. 11. (**g**) Control and *Lsd1* KO GV oocytes were cultured for 20 h in IBMX-containing medium with or without BN82002 (BN), as indicated, and the numbers of GV arrested (grey bar), MI (white bar), MII (black bar) and fragmented (yellow bar) oocytes were counted. The average proportion of oocytes at each stage from three experiments is plotted as a percentage of the total.

LSD1 plays an important role in the regulation of gene expression^15^,^22^,^31^,^32^,^33^. It is likely that the meiosis phenotype of *Lsd1* KO oocytes resulted from aberrant expression of essential genes. We therefore compared the transcriptomes of *Lsd1* KO and control GV oocytes by high-throughput RNA sequencing (RNA-seq) analysis. Using twofold change as a cutoff (FDR=0.01), 367 genes were upregulated and 252 genes were downregulated in *Lsd1* KO oocytes (Supplementary Fig. 5a). Consistent with the notion that LSD1 mainly represses gene expression, more genes were upregulated than downregulated in *Lsd1* KO oocytes (Supplementary Fig. 5a), and upregulated genes exhibited higher fold changes than downregulated genes (Supplementary Fig. 5b). Gene ontology (GO) term analysis of the misregulated genes revealed enrichment of pathways implicated in cell viability, embryonic development and reproductive system development, among others (Supplementary Fig. 5c). Notably, among the upregulated genes was *Cdc25b* (Supplementary Fig. 5d), which encodes a dual-specificity phosphatase that is essential for meiotic resumption by dephosphorylating and activating CDK1 (refs 8, 34). In agreement with the RNA-seq results, quantitative reverse transcription PCR (qRT–PCR) and western blot analyses confirmed the increases in *Cdc25b* transcript (∼2.7-fold) and protein (∼2.5-fold) levels in *Lsd1* KO oocytes, whereas the transcript level of *Wee2*, which encodes a kinase that antagonizes CDC25B by phosphorylating and inactivating CDK1 (refs 5, 30), was not altered (Fig. 4a,b). IHC and immunofluorescence (IF) analyses provided further evidence for CDC25B upregulation in *Lsd1* KO oocytes (Fig. 4c–e).

To determine whether excess CDC25B was responsible for precocious meiotic resumption, we assessed the impact of CDC25B inhibition. When *Lsd1* KO GV oocytes were cultured in the presence of BN82002, a CDC25 phosphatase inhibitor, the level of CDK1 phosphorylation (pY15-CDK1) was greatly restored, whereas the pY15-CDK1 level in control GV oocytes was not affected by BN82002, likely because CDK1 was already highly phosphorylated in these oocytes (Fig. 4f). Consistent with the restoration of CDK1 phosphorylation in BN82002-treated KO oocytes, enhanced GVBD was largely prevented (Fig. 4g). Collectively, our results supported the idea that precocious meiotic resumption in *Lsd1* KO oocytes was due to abnormal activation of CDK1, as a result of elevated CDC25B levels.

### Most *Lsd1* KO oocytes undergo apoptosis during meiosis I

**Figure 5:**
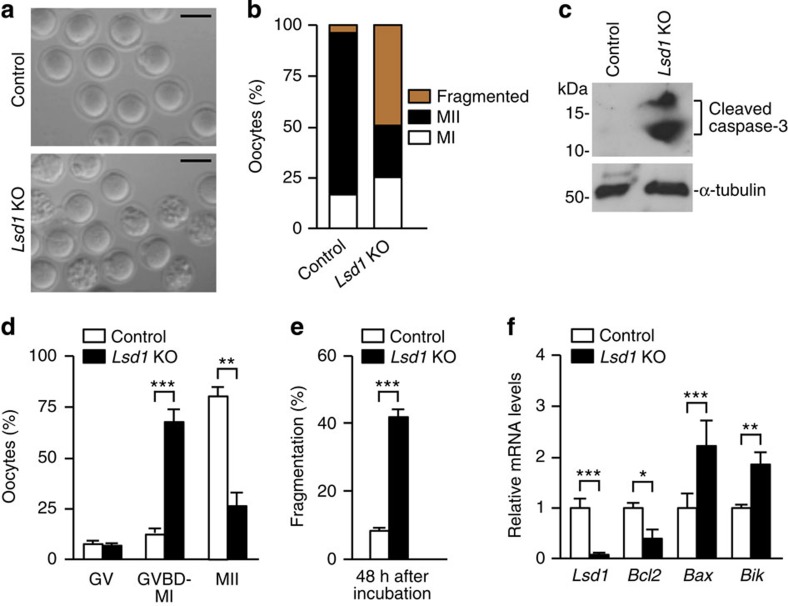
Most *Lsd1* KO oocytes are arrested at meiosis I and undergo apoptosis. (**a**,**b**) Oocytes collected from the oviducts of control and *Lsd1* KO mice 16 h post-hCG injection. (**a**) Representative images of the indicated groups. Scale bars, 80 μm. (**b**) The numbers of MI (white bar), MII (black bar) and fragmented (yellow bar) oocytes were counted, and the average proportion of oocytes at each stage from three experiments is plotted as a percentage of the total. (**c**) Oocytes collected from the oviducts of superovulated control and *Lsd1* KO mice were immunoblotted with antibodies specific for cleaved caspase-3 and α-tubulin (loading control). Each lane contained 150 oocytes. Full blots are provided in Supplementary Fig. 12. (**d**) GV oocytes were cultured in maturation medium for 12 h and then immunostained for α-tubulin and DNA to determine the meiotic stages. Shown are the percentages of GV, GVBD-MI, and MII oocytes (mean±s.e.m. from three experiments). Statistical comparisons of values were made using multiple *t*-test. **P<0.01; ***P<0.001. (**e**) GV oocytes were cultured in maturation medium for 48 h, and the percentages of fragmented oocytes are shown (mean±s.e.m. from three experiments). Statistical comparisons of values were made using unpaired *t*-test. ***P<0.001. (**f**) Quantitative RT–PCR analysis of *Lsd1, Bcl2, Bax* and *Bik* transcripts in GV oocytes from control and *Lsd1* KO mice. Data are presented as the mean±s.e.m. from three experiments. Statistical comparisons of values were made using multiple *t*-test. *P<0.05; **P<0.01; ***P<0.001.

The defect in sustaining prophase I arrest could contribute to, but not completely explain, the infertile phenotype of *Lsd1* KO female mice. The fact that KO mice produced relatively normal number of GV oocytes but substantially fewer MII oocytes (Supplementary Fig. 3b) suggested a major meiotic block following GVBD. We set out to determine whether LSD1 loss affected meiotic progression. After superovulation, the vast majority (∼80%) of oocytes collected from the oviducts of control mice had a clear polar body (arrested at the MII stage), whereas only a small fraction (∼25%) of *Lsd1* KO oocytes did. Rather, a large fraction (∼50%) of KO oocytes was fragmented (Fig. 5a,b). These fragmented oocytes exhibited features of apoptosis, including cytoplasm condensation, membrane protuberances and membrane-enclosed vesicles (Fig. 5a). Indeed, cleaved caspase-3, an indicator of apoptosis, was readily detected in oocytes collected from the oviducts of superovulated *Lsd1* KO mice (Fig. 5c). Furthermore, terminal deoxynucleotidyl transferase dUTP nick end labelling (TUNEL) assays revealed significantly more TUNEL-positive oocytes in *Lsd1* KO ovaries, as compared with their control counterparts (Supplementary Fig. 6a,b). Notably, most fragmented oocytes lacked an obvious polar body (Fig. 5a). Taken together, these results suggested that although *Lsd1* KO oocytes readily underwent GVBD and resumed meiosis, many of them failed to complete meiosis I and underwent apoptosis.

To confirm the timing of meiotic defects, GV oocytes were cultured in maturation medium and meiotic stages were determined by staining the spindles (α-tubulin) and DNA (DAPI). After 12 h of culture, ∼80% of control oocytes proceeded to the MII stage, as expected. In contrast, the majority (∼65%) of *Lsd1* KO oocytes failed to extrude the first polar body and was arrested at various phases of meiosis I, and only ∼26% completed meiosis I (Fig. 5d). These results further indicated that most *Lsd1* KO oocytes experienced a meiotic block before the completion of meiosis I.

To address whether LSD1 loss simply caused a delay in meiotic maturation, we cultured GV oocytes for 48 h. As shown in Fig. 5e, a large fraction (>40%) of *Lsd1* KO oocytes underwent fragmentation following prolonged culture. Collectively, our results indicated that LSD1 loss ultimately led to oocyte apoptosis, mostly at meiosis I, which likely accounts for the significant reduction in MII oocytes (Supplementary Fig. 3b).

While prolonged meiotic arrest could lead to oocyte apoptosis, dysregulation of genes involved in cell survival could also have contributed to the phenotype. Although *Lsd1* KO GV oocytes were morphologically normal, RNA-seq analysis revealed that differentially expressed genes included those involved in cell death and apoptosis (Supplementary Fig. 5c). qRT–PCR analysis confirmed that *Lsd1* KO GV oocytes indeed had an increased abundance of the proapoptotic transcripts *Bax* and *Bik* and a decreased abundance of the antiapoptotic transcript *Bcl-2* (Fig. 5f). Thus, *Lsd1* KO oocytes were likely prone to apoptosis.

### *Lsd1* KO oocytes exhibit chromosome defects

**Figure 6:**
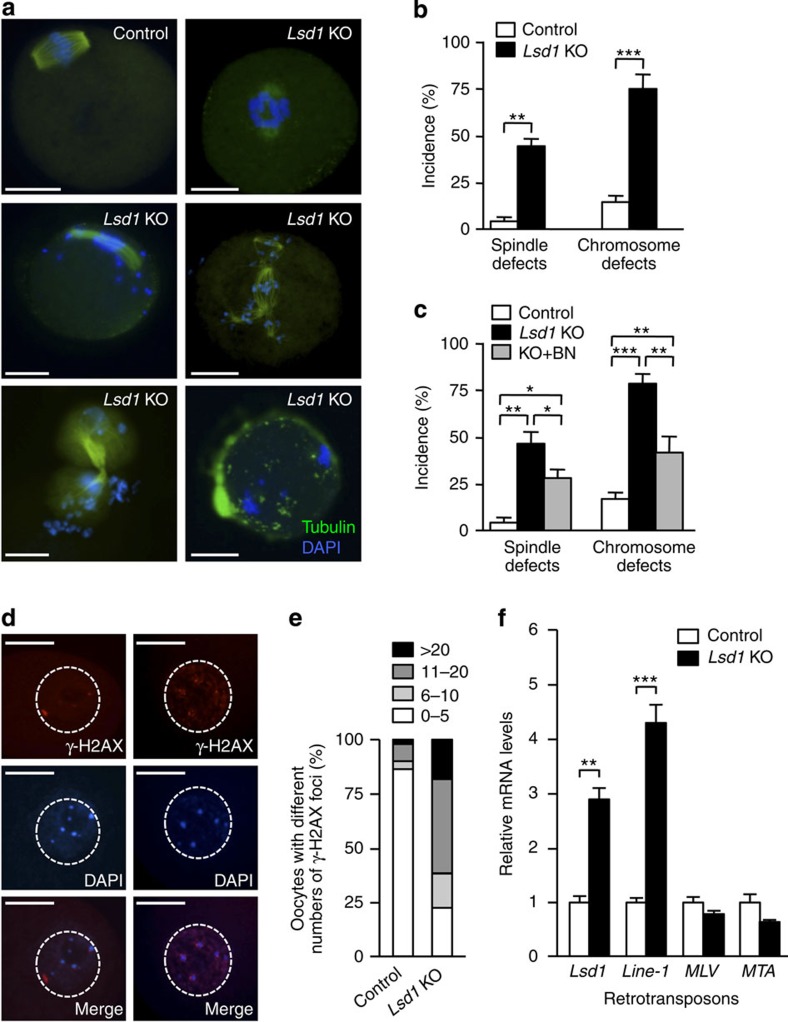
*Lsd1* KO oocytes exhibit chromosomal defects and DNA damage. (**a**,**b**) GV oocytes were collected from the ovaries of PMSG-primed females, cultured in maturation medium for 6 h, and then immunostained for α-tubulin (green) and DNA (blue) to examine spindle morphology and chromosome alignment. (**a**) Representative IF images showing normal spindle and chromosome structures in control oocytes and common abnormalities in *Lsd1* KO oocytes. Scale bars, 25 μm. (**b**) Frequencies of spindle and chromosomal abnormalities in control and *Lsd1* KO oocytes (mean±s.e.m. from three experiments). Statistical comparisons of values were made using multiple *t*-test. **P<0.01; ***P<0.001. (**c**) Effect of CDC25 inhibition on spindle and chromosome phenotypes in *Lsd1* KO oocytes. GV oocytes were collected and cultured in maturation medium for 1 h to induce GVBD and then further cultured either with or without the CDC25 phosphatase inhibitor BN82002 (BN) for 6 h. Shown are frequencies of abnormal spindle and chromosome defects in oocytes of the indicated groups (mean±s.e.m. from three experiments). Statistical comparisons of values were made using one-way ANOVA. *P<0.05; **P<0.01; ***P<0.001. (**d**,**e**) GV oocytes were examined for DNA double-strand breaks (DSBs) with anti-γ-H2AX staining. (**d**) Representative γ-H2AX (red), DAPI (blue) and merged images of control (left) and *Lsd1* KO (right) oocytes. The nuclei are circled. Scale bars, 25 μm. (**e**) The proportions of oocytes with various numbers of γ-H2AX foci in the nuclei are shown. (**f**) Quantitative RT–PCR analysis of retrotransposon transcripts in control and *Lsd1* KO oocytes. *IAP*, intracisternal A particles; *Line-1*, long interspersed nuclear element-1; *MLV*, murine leukaemia virus; *MTA*, mouse transposon A. Statistical comparisons of values were made using multiple *t*-test. **P<0.01; ***P<0.001.

To further characterize the meiotic defects, we cultured GV oocytes in maturation medium for 6 h and carefully examined the spindle and chromosome structures. While most control oocytes proceeded through meiosis I normally, *Lsd1* KO oocytes frequently harboured abnormal spindles with misaligned and lagging chromosomes. Among all KO oocytes examined, ∼40% had clear abnormalities in spindle organization and ∼75% exhibited obvious defects in chromosome congression, alignment and/or segregation (Fig. 6a,b). We also examined kinetochore-microtubule attachment by double immunostaining with anti-α-tubulin (microtubule) and CREST antisera^35^, which stain centromeres, where kinetochores assemble. Consistent with the spindle and chromosomal abnormalities described above, kinetochores and microtubules were frequently dissociated in *Lsd1* KO oocytes (Supplementary Fig. 7).

CDC25B has been implicated in spindle formation during mitosis and meiosis^36^,^37^. IF analysis revealed that CDC25B was accumulated in the spindle apparatus of oocytes at MI and MII stages (Supplementary Fig. 8), consistent with previous reports^9^,^10^. We therefore asked whether increased CDC25B levels in *Lsd1* KO oocytes contributed to the spindle abnormalities during meiosis. When *Lsd1* KO GV oocytes were cultured in the presence of the CDC25 inhibitor BN82002, the frequencies of abnormal spindle and chromosomal defects were significantly decreased, although the defects were not fully prevented (Fig. 6c). These results suggested that CDC25B accumulation partly contributed to the spindle and chromosomal abnormalities observed in *Lsd1* KO oocytes, although we cannot completely rule out the possibility that the partial effect was due to incomplete inhibition of CDC25B by BN82002 (Fig. 4f).

A previous report showed that disruption of the H3K4 methyltransferase gene *Prdm9* (also known as *Meisetz*) in mice results in infertility in both sexes due to severe impairment of the double-strand break (DSB) repair pathway^12^, suggesting that maintenance of appropriate H3K4 methylation levels is important for genome integrity in germ cells. LSD1 depletion in oocytes led to a substantial increase in global H3K4me2 (Fig. 1b), which may cause genomic instability. We measured DNA DSBs in GV oocytes by the presence of phosphorylated histone H2AX (γ-H2AX) and found that the number of DSBs in *Lsd1* KO oocytes was substantially elevated compared with control oocytes (Fig. 6d,e). LSD1 depletion also led to increased levels of transcripts for some retrotransposons, including intracisternal A particles (*IAP*) and long interspersed nuclear element-1 (*Line-1*; Fig. 6f), suggesting that LSD1 is required for their suppression in oocytes. Retrotransposon derepression may have contributed to the increased DNA damage observed in *Lsd1* KO oocytes, as retrotransposon activation and DSBs are often correlated^38^. The causal relationship between chromosome abnormalities and aberrant spindle structures remains to be determined. It has been well established that chromosome integrity and dynamics and spindle formation depend on each other during meiosis^39^,^40^. Given that some oocytes exhibited chromosome defects without obvious spindle aberrations in *Lsd1* KO oocytes (Fig. 6a,b), it is tempting to speculate that both abnormal spindles and DNA damage contributed to the chromosome defects, which likely played an important part in inducing meiotic block.

### *Lsd1* KO MII oocytes are mostly aneuploid and unfertilizable

**Figure 7:**
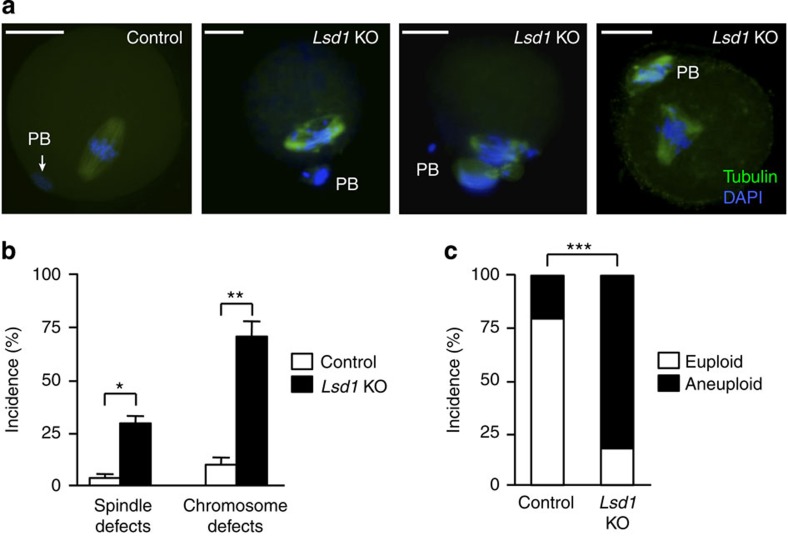
*Lsd1*-null MII oocytes exhibit chromosomal defects and aneuploidy. (**a**,**b**) Oocytes were collected from the oviducts 16 h post-hCG injection and then immunostained for α-tubulin (green) and DAPI (blue) to examine spindle morphology and chromosome alignment. (**a**) Representative IF images showing normal spindle and chromosome structures in control MII oocytes and common abnormalities in *Lsd1* KO MII oocytes. PB, polar body. Scale bars, 25 μm. (**b**) Frequencies of abnormal spindle and chromosome defects in MII oocytes of the indicated groups (mean±s.e.m. from three experiments). Statistical comparisons of values were made using multiple *t*-test. *P<0.05; **P<0.01. (**c**) Chromosome spreads of MII oocytes from control and *Lsd1* KO mice were performed, and chromosome numbers counted. Shown are the percentages of euploid (20 chromosomes) and aneuploid (fewer or more than 20 chromosomes) MII oocytes from control and *Lsd1* KO mice. Statistical comparisons of values were made using unpaired *t*-test. ***P<0.001.

Although most *Lsd1* KO oocytes experienced a meiotic block and underwent apoptosis during meiosis I, some of them completed meiosis I and developed to the MII stage (Fig. 5a–d). However, IF analysis revealed that the majority (∼70%) of *Lsd1* KO MII oocytes had chromosome defects and a substantial fraction (∼30%) also exhibited abnormal spindle organization (Fig. 7a,b), similar to KO oocytes at meiosis I (Fig. 6a,b). Conceivably, misalignment and missegregation events during meiosis I could cause aneuploidy, which is a major cause of infertility^39^,^41^. Indeed, *Lsd1* KO MII oocytes showed a markedly increased aneuploidy rate (>80%), relative to MII oocytes from control mice (∼20%) (Fig. 7c).

**Figure 8:**
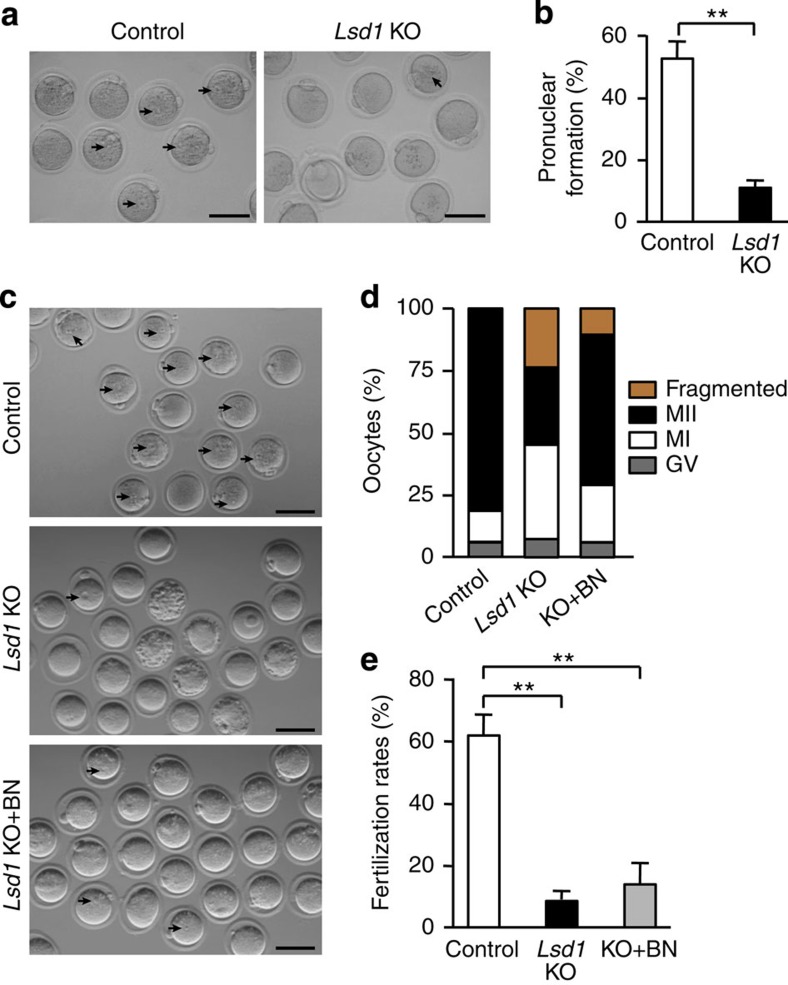
*Lsd1*-null MII oocytes are mostly unfertilizable. (**a**,**b**) Morphologically ‘normal' MII oocytes isolated from superovulated control and *Lsd1* KO mice were activated with strontium chloride and scored for pronuclear (PN) formation after 7 h. (**a**) Representative images of the oocytes following strontium chloride exposure. Arrows indicate the pronuclei. Scale bars, 80 μm. (**b**) The percentages of oocytes with pronuclei are shown (mean±s.e.m. of three independent experiments). Statistical comparisons of values were made using unpaired *t*-test. **P<0.01. (**c**–**e**) Fully grown GV oocytes collected from control and *Lsd1* KO mice were incubated in maturation medium for 1 h to induce GVBD and then further cultured for 24 h with or without the CDC25 phosphatase inhibitor BN82002 (BN), as indicated. After determining the meiotic stages, the oocytes were inseminated with sperm from wild-type males, and fertilization rates (judged by PN formation) were determined after 7 h. (**c**) Representative images of the oocytes 7 h after insemination. Arrows indicate the pronuclei. Scale bars, 80 μm. (**d**) The numbers of GV arrested (grey bar), MI (white bar), MII (black bar) and fragmented (yellow bar) oocytes were counted before insemination. The average proportion of oocytes at each stage from three experiments is plotted as a percentage of the total. (**e**) Fertilization rates. The numbers of fertilized eggs (those with pronuclei) were counted 7 h after insemination, and they were divided by the total numbers of MII oocytes to determine the fertilization rates (mean±s.e.m. from three experiments). Statistical comparisons of values were made using one-way ANOVA. **P<0.01.

We assessed the developmental competence of *Lsd1* KO MII oocytes that were morphologically ‘normal' by parthenogenetic activation^42^. Following strontium chloride exposure, control MII oocytes were efficiently activated, as evidenced by the formation of pronuclei. In contrast, most *Lsd1* KO MII oocytes failed to form pronuclei (Fig. 8a,b), suggesting that most *Lsd1* KO MII oocytes could not be fertilized. To further assess the fertilizability of *Lsd1* KO oocytes, we derived MII oocytes by culturing GV oocytes and performed *in vitro* fertilization experiments. After 24 h of culture, over 80% of control GV oocytes developed to MII oocytes, ∼60% of which were successfully fertilized by wild-type sperm, judged by pronuclear formation, whereas only ∼30% of *Lsd1* KO GV oocytes matured to the MII stage and considerable fractions were arrested at MI or fragmented, and the fertilization rate (among all MII oocytes) was <10% (Fig. 8c–e). To determine whether inhibition of CDC25B could improve oocyte maturation and fertilization rates, *Lsd1* KO GV oocytes were matured in the presence of the CDC25 phosphatase inhibitor BN82002 and then inseminated. While BN82002 markedly facilitated *Lsd1* KO oocyte maturation (∼60% developed to MII stage), the fertilization rate was not improved (Fig. 8c–e). Thus, even though a small number of *Lsd1* KO oocytes developed to MII oocytes, they were mostly unfertilizable, likely due to severe chromosomal defects.

## Discussion

**Figure 9:**
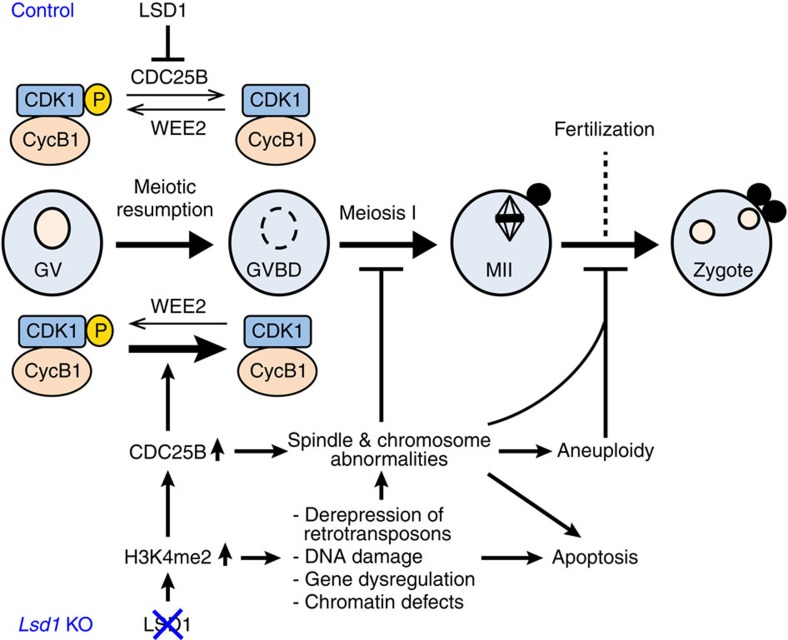
Proposed effects of LSD1 depletion on meiotic progression in female mice. LSD1 depletion in growing oocytes leads to elevation of global H3K4me2. One consequence is increased expression of CDC25B, which dephosphorylates and activates CDK1, resulting in precocious meiotic resumption. CDC25B upregulation also contributes to subsequent spindle and chromosomal abnormalities. Other consequences of H3K4me2 elevation, including derepression of retrotransposons, DNA DSBs, altered gene expression and chromatin defects also play important roles in inducing chromosomal abnormalities. Most *Lsd1*-null oocytes are arrested at meiosis I and undergo apoptosis, and the small number of oocytes that develop to MII oocytes have severe chromosomal and spindle defects and are mostly aneuploidy and unfertilizable.

Progression of meiosis is controlled by unique gene expression programs and involves marked chromatin remodelling. Epigenetic mechanisms, such as posttranslational modifications of histones, play crucial roles in gene expression and chromatin structure. While progress has been made in documenting changes in histone modifications during meiosis^14^, the functional significance of these changes and the key epigenetic regulators involved in meiosis (especially during female germ cell development) are poorly understood. In this study, we demonstrate that LSD1 controls global H3K4me2 levels in mouse oocytes and regulates the expression of CDC25B, a key component of the signalling pathway that governs meiotic progression. We provide evidence that upregulation of CDC25B in *Lsd1*-null oocytes, which leads to activation of CDK1, is largely responsible for precocious resumption of meiosis. Even though *Lsd1*-null oocytes readily undergo GVBD, they frequently exhibit spindle and chromosomal defects, with most of them being arrested at meiosis I and undergoing apoptosis. The small numbers of oocytes that survive and develop to the MII stage also exhibit high frequencies of chromosomal aberrations and aneuploidy, making these oocytes mostly unfertilizable. Our data suggest that upregulation of CDC25B partially contributes to the spindle and chromosomal abnormalities. Other consequences of LSD1 loss and H3K4me2 elevation, including derepression of retrotransposons, DNA damage, defects in chromosome dynamics, and dyregulation of other genes, likely also play important roles in chromosomal defects and apoptosis (Fig. 9).

It is well established that CDC25B activation plays a critical role in meiotic resumption^7^,^8^,^34^. In addition to its activity, CDC25B levels fluctuate during meiotic maturation. Specifically, CDC25B level is relatively low in GV oocytes, exhibits an elevation at GVBD, and is substantially reduced at metaphase I^9^,^10^. These observations suggest that regulation of CDC25B expression is an important mechanism that controls the timing of meiotic resumption. Although the regulatory mechanisms controlling CDC25B levels are not well understood, enhanced translation and protein degradation have been implicated in the dynamic changes of CDC25B levels at GVBD and metaphase I, respectively^9^. Our results indicate that LSD1 plays an essential role in keeping CDC25B below a threshold level in growing oocytes to maintain prophase I arrest. The drastic decrease of LSD1 in oocytes at the antral follicle stage, when meiotic competence is acquired, likely contributes to the accumulation of CDC25B required to induce meiotic resumption. Because loss of LSD1 results in increases in both *Cdc25b* transcript and protein, LSD1 likely represses *Cdc25b* transcription. While the paucity of material makes it difficult to assess whether the *Cdc25b* gene is a direct target of LSD1 in oocytes, chromatin immunoprecipitation followed by high-throughput sequencing (ChIP-seq) analysis of embryonic stem cells has revealed LSD1 binding at the promoter region of *Cdc25b*^43^,^44^, suggesting direct regulation of *Cdc25b* transcription by LSD1.

Normally, once meiotic resumption is induced, CDC25B level quickly decreases, likely due to proteasome-mediated degradation^9^. However, in *Lsd1*-null oocytes, high levels of CDC25B persists throughout all stages of meiosis, suggesting that CDC25B degradation may also be impaired. Interestingly, RNA-seq and qRT–PCR analyses reveal that *Cdh1* is downregulated in *Lsd1*-null oocytes. CDH1 (also known as FZR) is a cofactor of the anaphase-promoting complex/cyclosome (APC/C), an E3 ubiquitin ligase that targets specific substrates, including cyclin B1 and CDC25B, for degradation^45^,^46^. Despite the marked activation of CDK1 and reduced CDH1 levels, we did not observe any pronounced increase in cyclin B1 levels in *Lsd1*-null oocytes (Fig. 3a). Nevertheless, it is possible that decreased APC/C activity, as a result of CDH1 reduction, has contributed to the failure to degrade CDC25B after GVBD. Given the enrichment of CDC25B in the spindle apparatus during meiosis (Supplementary Fig. 8) and the previous reports implicating the involvement of CDC25B in spindle formation during mitosis and meiosis^36^,^37^, failure to degrade CDC25B after GVBD may have played a role in spindle aberrations observed in *Lsd1*-null oocytes. Consistent with this notion, treatment of *Lsd1*-null oocytes with a CDC25 inhibitor partially rescues the spindle and chromosomal defects and facilitates meiotic maturation (Figs 6c and 8c,d).

In addition to regulating gene expression, LSD1 is likely a key factor that regulates chromatin structure. We show that depletion of LSD1 in oocytes results in a substantial increase in global H3K4me2 (Fig. 1b) and concomitantly, increased histone acetylation (Supplementary Fig. 2b), implying a generally open chromatin state. While chromatin condensation (to form chromosomes) occurs at GVBD in *Lsd1*-null oocytes, the chromosomes may not be completely normal and thus may affect subsequent meiotic progression. The open chromatin state likely has also contributed to increased DNA damage and derepression of retrotransposons observed in *Lsd1*-null oocytes.

## Methods

### Mice

Experimental mice were maintained on a mixed C57BL/6 and 129 background. They were used in accordance with the National Institutes of Health Guide for the Care and Use of Laboratory animals, with Institutional Care and Use Committee-approved protocols at The University of Texas MD Anderson Cancer Center (MDACC). *Lsd1*^*fl/fl*^/*Zp3-Cre*^*+*^ (*Lsd1* KO) mice and *Lsd1*^*fl/+*^/*Zp3-Cre*^*−*^ (control) littermates were produced by crossing mice bearing the *Lsd1*^*fl*^ allele^24^ with *Zp3-Cre* transgenic mice (Supplementary Fig. 2a). Mice were genotyped by PCR using genomic tail DNA (Supplementary Fig. 2b). All primers used for genotyping and other applications are listed in Supplementary Table 1.

### Oocyte maturation and parthenogenetic activation

Fully grown GV oocytes were obtained from the ovaries of 4–6-week-old female mice 48 h after intraperitoneal injection of 5 IU of PMSG (Sigma). Ovaries were placed in a Petri dish with prewarmed (37 °C) M2 medium (Invitrogen) supplemented with IBMX (Sigma) so as to prevent oocytes from undergoing GVBD. GV oocytes were released by puncturing antral follicles with a fine needle on the stage of a dissecting microscope. To obtain MII oocytes, 5 IU of human chorionic gonadotrophin (hCG, Sigma) was administered 48 h after PMSG injection. Mice were euthanized 16 h after hCG injection, and oocytes were collected from the oviducts and released into a hyaluronidase/M2 solution for removal of the cumulus cells. For *in vitro* maturation, oocytes were washed and cultured in IBMX-free M16 medium (Millipore) for various periods of time at 37 °C in 5% CO_2_ atmosphere. Parthenogenetic activation of MII oocytes was achieved by exposing oocytes into Ca^2+^-free media containing 10 mM strontium chloride (SrCl_2_, Sigma) for 7 h (ref. 42).

### *In vitro* fertilization

Epididymis was dissected into prewarmed (37 °C) human tubal fluid (HTF). Four microlitre of fresh sperm were added to a 200-μl HTF drop covered with mineral oil and capacitated for 2 h in the incubator before adding oocytes. The oocytes were added directly to the sperm suspension and incubated for 7 h at 37 °C, 5% CO_2_ in HTF.

### Embryo collection

Mice were superovulated and fertilized by wild-type males, and checked for the presence of vaginal plugs. E0.5 embryos (zygotes) were collected from the oviducts and released into a hyaluronidase/M2 solution for dissociation. E1.5 (2-cell) embryos were flushed out of the infundibula of the oviducts, and E3.5 embryos (blastocysts) were flushed out of the uteri.

### Histological analysis

Ovaries were collected and fixed in formalin overnight, processed, and embedded in paraffin by the Pathology Core Services Facility at MDACC using standard protocols. Ovaries were serially sectioned at 5 μm and stained with hematoxylin and eosine (H&E) or with periodic acid-Schiff (PAS)-haematoxylin. IHC was performed using standard protocols. The antibodies used are listed in Supplementary Table 2.

### Immunofluorescence

Oocytes were fixed in 4% paraformaldehyde in PBS for 30 min at room temperature and permeabilized for 15 min in 0.1% Triton X-100 in PBS at room temperature. Antibody staining was performed using standard protocols. The antibodies used are listed in Supplementary Table 2.

### Western blot analysis

Hundred to 150 oocytes were collected, washed in PBS containing 1% polyvinylpyrrolidine (PVP) and frozen in SDS sample buffer. Western blot was performed using standard protocols. The antibodies used are listed in Supplementary Table 2.

### Chromosome spreads

Oocytes were placed in hypotonic solution (1% sodium citrate) for 20 min and fixed by methanol: glacial acetic acid (3:1). Chromosome spreads were visualized with Giemsa staining.

### Quantitative RT–PCR

Total RNA was extracted from oocytes using the PicoPure RNA Isolation Kit (Life Technologies) according to the manufacturer's instruction, followed by reverse transcription (RT) using Superscript RT kit (Bio-Rad) to generate cDNA libraries. Quantitative RT–PCR was performed using iTaq Universal SYBR Green Supermix with ABI 7900 Real-Time PCR system (Applied Biosystems) using primers for the following genes and retrotransposons: *Lsd1* (NM_133872), *Cdc25b* (NM_001111075), *Wee2* (NM_201370), *Bcl2* (NM_009741), *Bax* (NM_007527), *Bik* (NM_007546), *IAP*, *Line-1*, *MLV*, and *MTA*. The primers used for quantitative RT–PCR analyses are listed in Supplementary Table 1.

### RNA-seq analysis

For RNA-seq analysis, three biological replicates were prepared for each genotype. Each sample, which contained 100 GV oocytes collected from 2–3 mice, was lysed directly in 1 μl of prelude direct lysis buffer (Nugen). RNA was then subject to amplification using the ovation RNA-seq system v2 (Nugen). Amplified cDNA was fragmented using Covaris and paired-end libraries were then constructed using the TruSeq RNA Sample Preparation Kit v2 (Illumina) starting from end repair. The libraries were sequenced using a 2 × 76 bases paired end protocol on the Illumina HiSeq 2000 instrument, generating 20–27 million pairs of reads per sample. Each pair of reads represents a cDNA fragment from the library. The reads were mapped to mouse genome (mm9) by TopHat2 (ref. 47). The overall mapping rate is 60–90%. The number of fragments in each known gene from RefSeq database^48^ (downloaded from UCSC Genome Browser) was enumerated using htseq-count from HTSeq package (version 0.5.3p9, http://www-huber.embl.de/users/anders/HTSeq/). The differential expression between conditions was statistically accessed by R/Bioconductor package DESeq^49^. Genes with false discovery rate≤0.01 and fold change ≥2 were called significant. Gene clustering and heatmap were done by Cluster 3.0 and TreeView. GO analysis was performed using Ingenuity Pathway Analysis (IPA) software.

### Statistical analysis

Data were collected from at least three independent experiments unless otherwise specified. Values were analysed by one-way ANOVA, unpaired *t*-test or multiple *t*-test, and *P*<0.05 was considered statistically significant. The *P* values for GO analysis were calculated using the right-tailed Fisher's Exact Test by IPA.

## Additional information

**Accession codes:** The RNA-seq data have been deposited in the GEO database under accession code GSE73803.

**How to cite this article:** Kim, J. *et al*. LSD1 is essential for oocyte meiotic progression by regulating CDC25B expression in mice. *Nat. Commun.* 6:10116 doi: 10.1038/ncomms10116 (2015).

## Supplementary Material

Supplementary Information Supplementary Figures 1-13 and Supplementary Tables 1-2
