# Corrigendum: LSD1 is essential for oocyte meiotic progression by regulating CDC25B expression in mice

**DOI:** 10.1038/ncomms11467

**Published:** 2016-04-20

**Authors:** Jeesun Kim, Anup Kumar Singh, Yoko Takata, Kevin Lin, Jianjun Shen, Yue Lu, Marc A. Kerenyi, Stuart H. Orkin, Taiping Chen

Nature Communications 6: Article number: 10116 10.1038/ncomms10116 (2015); Published: 12022015; Updated: 04202016

In Fig. 6f of this article, the histogram label ‘Lsd1' should read ‘IAP'. The correct version of Fig. 6 appears below.


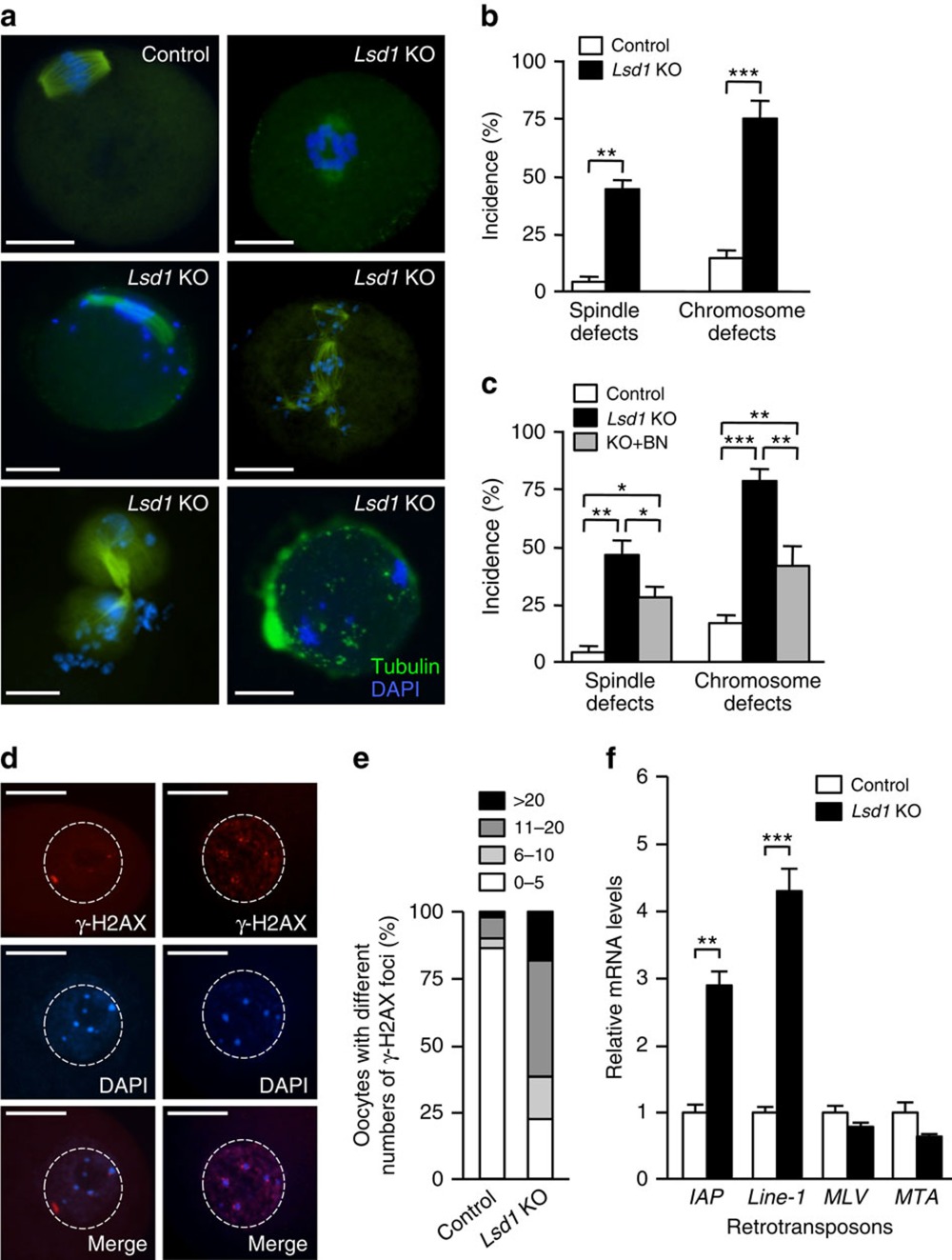


**Figure 6**

