# Supplementary material for: LSD1 is essential for oocyte meiotic progression by regulating CDC25B expression in mice
Source: Nat Commun. 2015 Dec 2;6:10116. doi: 10.1038/ncomms10116 (PMC4686821; doi:10.1038/ncomms10116)
Supplement: Supplementary Information — Supplementary Figures 1-13 and Supplementary Tables 1-2 [file ncomms10116-s1.pdf]

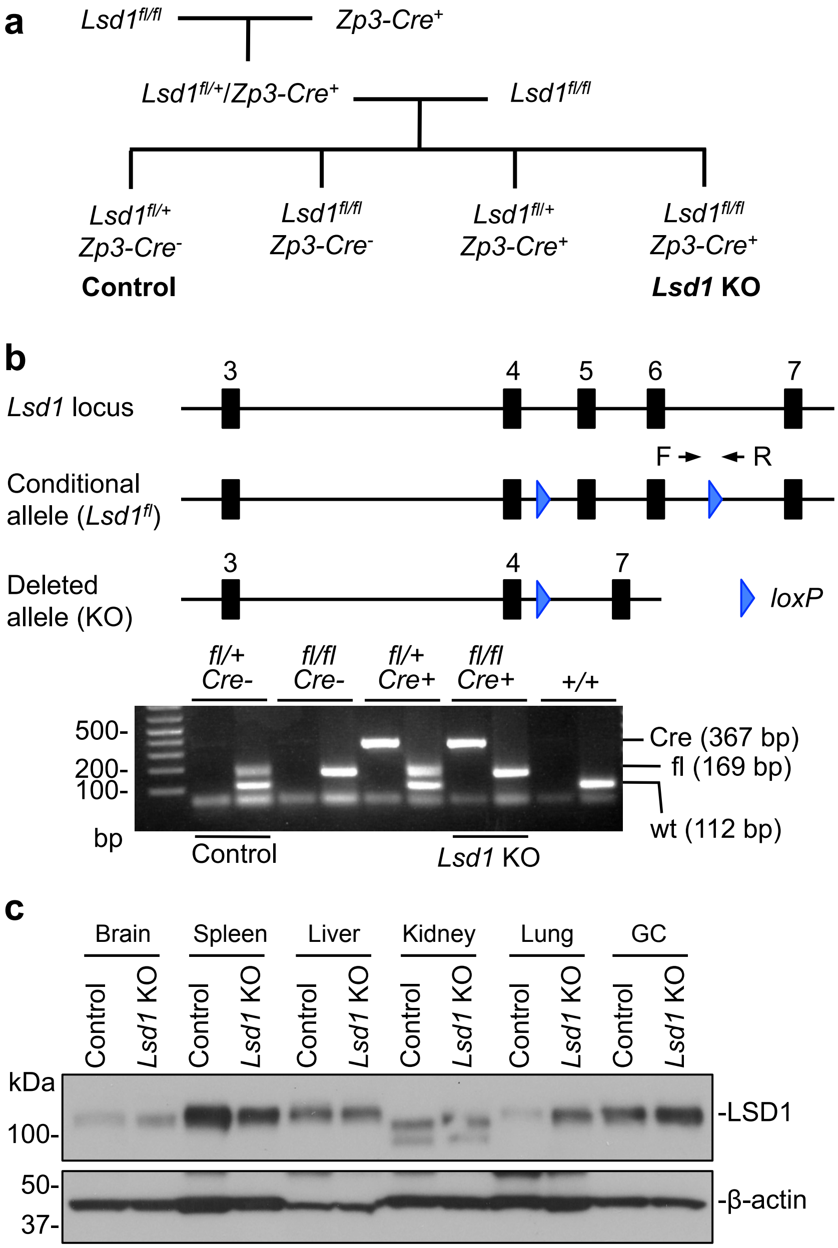

Supplementary Figure 1. Conditional deletion of *Lsd1* in growing oocytes using *Zp3*-Cre

(**a**) Mating scheme for producing *Lsd1* knockout (KO) and control mice. (**b**) Genotyping strategy. The locations of the forward (F) and reverse (R) primers used for PCR are indicated. Amplification of the wild-type and *Lsd1* conditional alleles with the primers yields 112-bp and 169-bp fragments, respectively. Representative genotyping results using tail-tip genomic DNA are shown. For each sample, the left lane is *Cre* PCR, and the right lane is *Lsd1* PCR. (**c**) Western blot analysis of LSD1 in somatic tissues. β-actin serves as a loading control. GC, granulosa cells. Full blots are provided in Supplementary Figure 13.

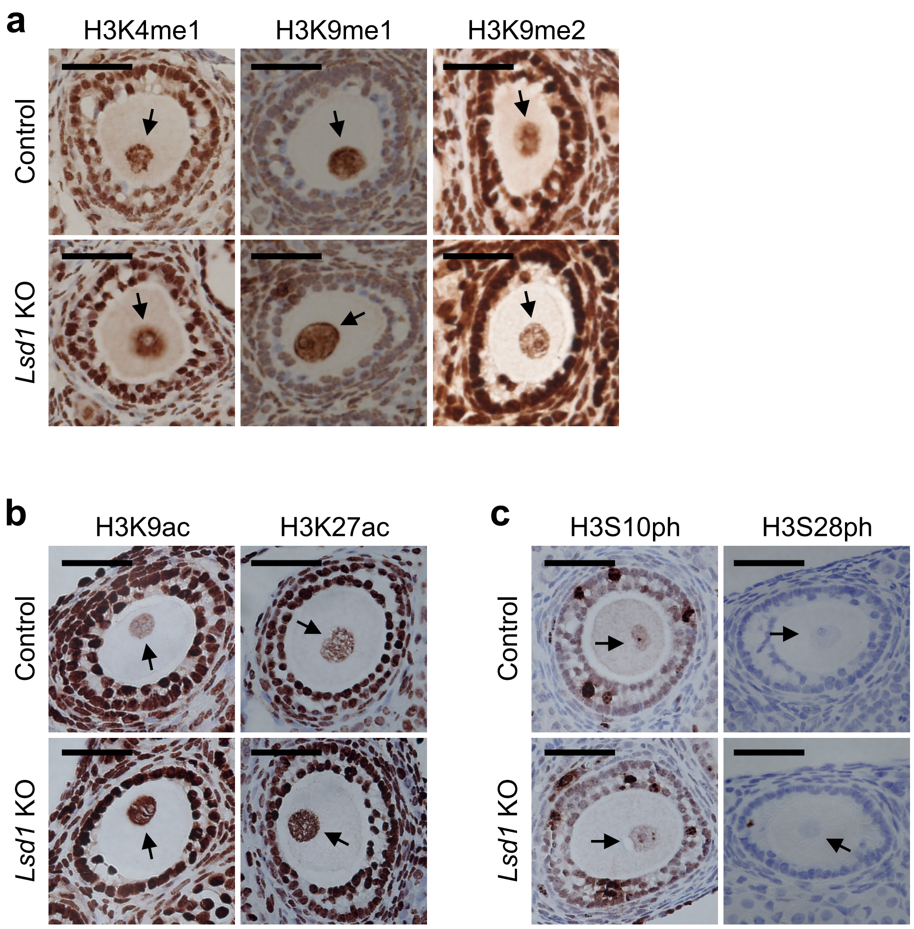

Supplementary Figure 2. The levels of various histone marks in control and *Lsd1* KO oocytes

Immunohistochemical (IHC) analyses of ovarian sections using antibodies specific for (**a**) histone methylation (H3K4me1, H3K9me1, H3K9me2), (**b**) acetylation (H3K9ac, H3K27ac), and (**c**) phosphorylation (H3S10ph, H3S28ph) marks, as indicated. Representative images of secondary follicles are shown. The oocyte nuclei are indicated by arrows. Scale bar, 40 μm.

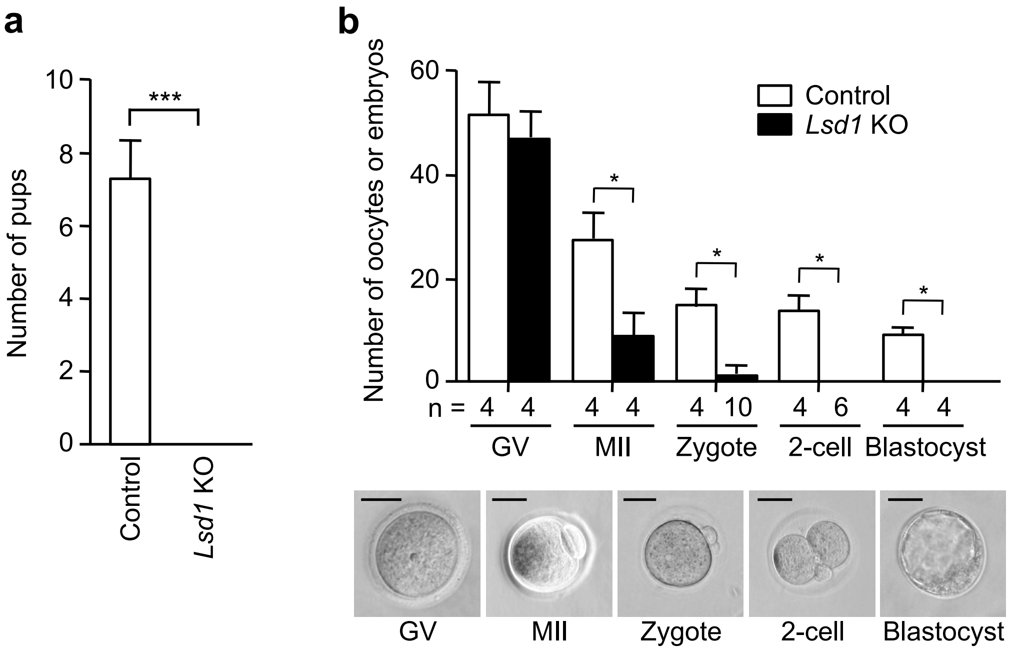

Supplementary Figure 3. *Lsd1* KO female mice are infertile

(**a**) 2 month-old control and *Lsd1* KO females were crossed with wild-type males, and the average number of pups per litter delivered by the females were recorded. Shown are the results (mean ± SEM) from 2 control (8 litters) and 4 KO mothers. ***P<0.001 (Student's *t*-test). (**b**) The numbers (mean ± SEM) of germinal vesicle (GV) and metaphase II (MII) oocytes and zygote-, 2-cell-, and blastocyst-stage embryos obtained from control and *Lsd1* KO females. The numbers (n) of female mice used to obtain the data are indicated. *P<0.05 (Student's *t*-test). Fully-grown GV oocytes were collected from the ovaries of pregnant mare's serum gonadotrophin (PMSG)-primed mice (4-6 weeks of age). MII oocytes were harvested from the oviducts at 16 hours after human chorionic gonadotrophin (hCG) injection. To obtain zygotes, 2-cell embryos, and blastocysts, females were injected with hCG and mated with wild-type males, and embryos were harvested at 24 h, 48 h, and 96 h post-injections, respectively. Zygotes were collected from the oviducts, 2-cell embryos were flushed out the infundibula of the oviducts, and blastocystes were flushed out of the uteri. Representative images of oocytes and embryos at different stages are shown. Scale bars, 40 μm.

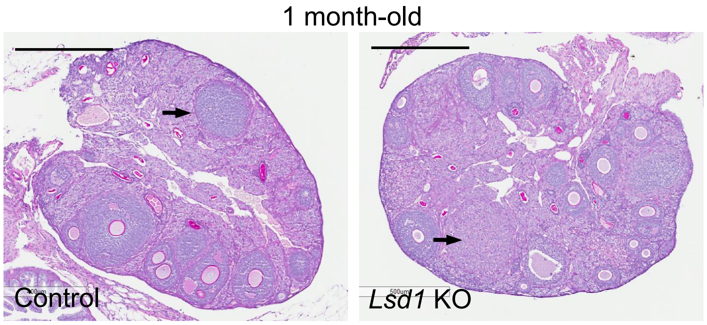

Supplementary Figure 4. *Lsd1* KO ovaries are normal at 1 month of age
Periodic acid-Schiff (PAS)-hematoxylin staining showing the histopathological features of ovaries from 1 month-old control and *Lsd1* KO mice. Arrows indicate corpus lutea. Scale bars, 500 μm.

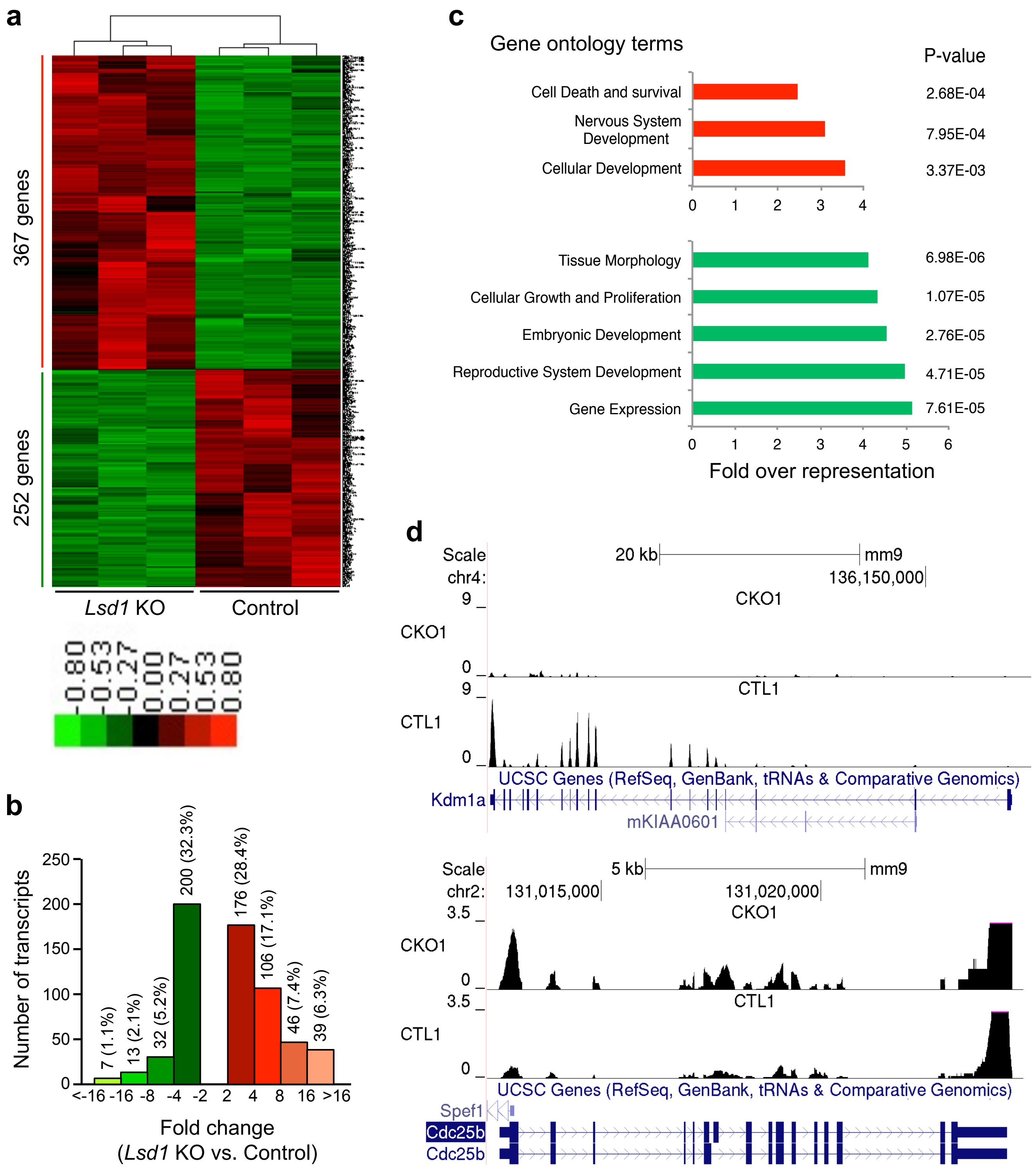
a
367 genes
252 genes
Lsd1 KO
Control
-0.80
-0.53
-0.27
0.00
0.27
0.53
0.80
b
Number of transcripts
250
200
150
100
50
0
7 (1.1%)
13 (2.1%)
32 (5.2%)
200 (32.3%)
176 (28.4%)
106 (17.1%)
46 (7.4%)
39 (6.3%)
<-16 -16 -8 -4 -2 2 4 8 16 >16
Fold change
(Lsd1 KO vs. Control)
c
Gene ontology terms
P-value
Cell Death and survival
2.68E-04
Nervous System Development
7.95E-04
Cellular Development
3.37E-03
0 1 2 3 4
Tissue Morphology
6.98E-06
Cellular Growth and Proliferation
1.07E-05
Embryonic Development
2.76E-05
Reproductive System Development
4.71E-05
Gene Expression
7.61E-05
0 1 2 3 4 5 6
Fold over representation
d
Scale
20 kb
mm9
chr4:
136,150,000
9
CKO1
0
CTL1
UCSC Genes (RefSeq, GenBank, tRNAs & Comparative Genomics)
Kdm1a
mKIAA0601
Scale
5 kb
mm9
chr2:
131,015,000
131,020,000
3.5
CKO1
0
CTL1
UCSC Genes (RefSeq, GenBank, tRNAs & Comparative Genomics)
Spef1
Cdc25b
Cdc25b

Supplementary Figure 5. Transcriptional profiling of GV oocytes by RNA-seq
(**a**) Heat map of relative expression levels of differentially expressed genes in *Lsd1* KO compared to control GV oocytes. Expression analysis was performed in triplicate on 100 GV oocytes per sample. For each gene, expression is shown relative to the average expression level of the gene across all samples. Green and red represent lower-than-average and higher-than-average expression levels, respectively. (**b**) Distribution of differentially expressed genes at various magnitudes of difference in expression levels between *Lsd1* KO and control oocytes detected by RNA-Seq analysis. The number and the percentage of each group of genes in total changed genes are indicated above each bar. (**c**) Gene ontology (GO) analysis of up-regulated (red) or down-regulated (green) genes in *Lsd1* KO oocytes using Ingenuity Pathway Analysis (IPA) software. Fold over representation indicates the observed percentage of misregulated genes in a particular GO category over the percentage expected on the basis of all GO-annotated genes. The P values were calculated using the right-tailed Fisher Exact Test by IPA. (**d**) Genome browser screenshots of *Lsd1* (*Kdm1a*) and *Cdc25b* loci showing RNA-seq data for control (CTL) and *Lsd1* KO (CKO).

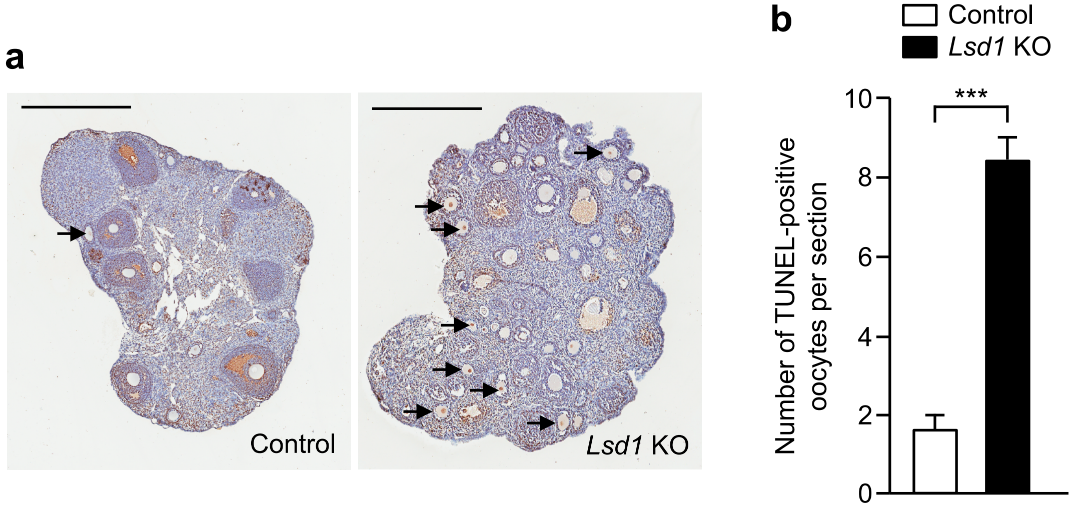

Supplementary Figure 6. TUNEL assays of control and *Lsd1* KO ovaries
IHC analysis of ovaries from 2 month-old control and *Lsd1* KO mice using a TUNEL assay kit. (**a**) IHC images. Arrows indicate TUNEL-positive oocytes (brown signal). Scale bars, 500 µm. (**b**) Quantification of TUNEL-positive oocytes. For each genotype, 5 sections were examined. The numbers of TUNEL-positive oocytes per section (mean ± SEM) are shown. ***P<0.001 (Student's *t*-test).

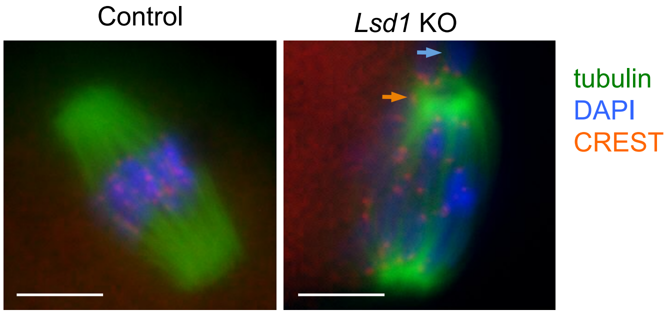

Supplementary Figure 7. LSD1-depleted oocytes frequently exhibit defects in kinetochore-microtubule attachment

Control and *Lsd1* KO GV oocytes were cultured in maturation medium for 6 hours and immunostained for α-tubulin (green), DAPI (blue), and CREST (red). Representative images are shown. The blue arrow indicates a misaligned chromosome and the orange arrow indicates a kinetochore that is not associated with microtubules. Scale bars, 10 μm.

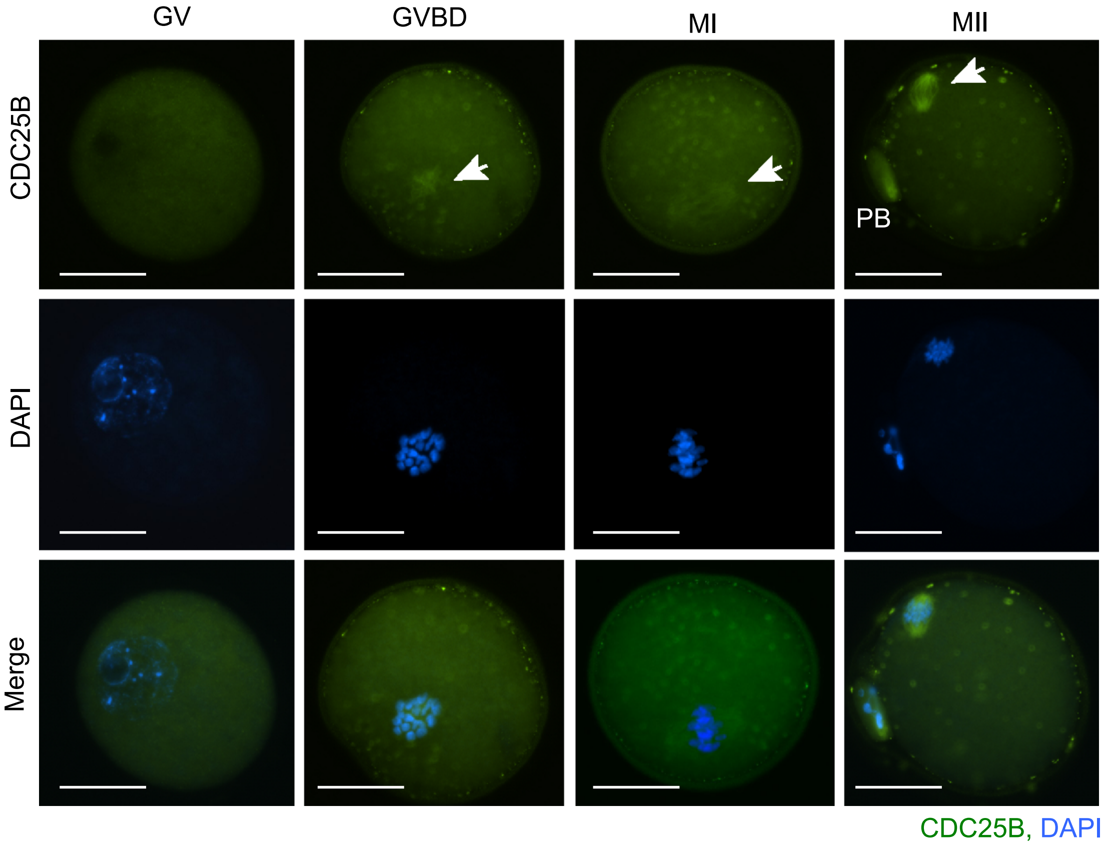

Supplementary Figure 8. CDC25B localization during meiotic progression
Immunofluorescence microscopy of wild-type oocytes stained for CDC25B (green) and DNA (blue). White arrows indicate CDC25B localization at spindle. PB, polar body. Scale bars, 25 μm.

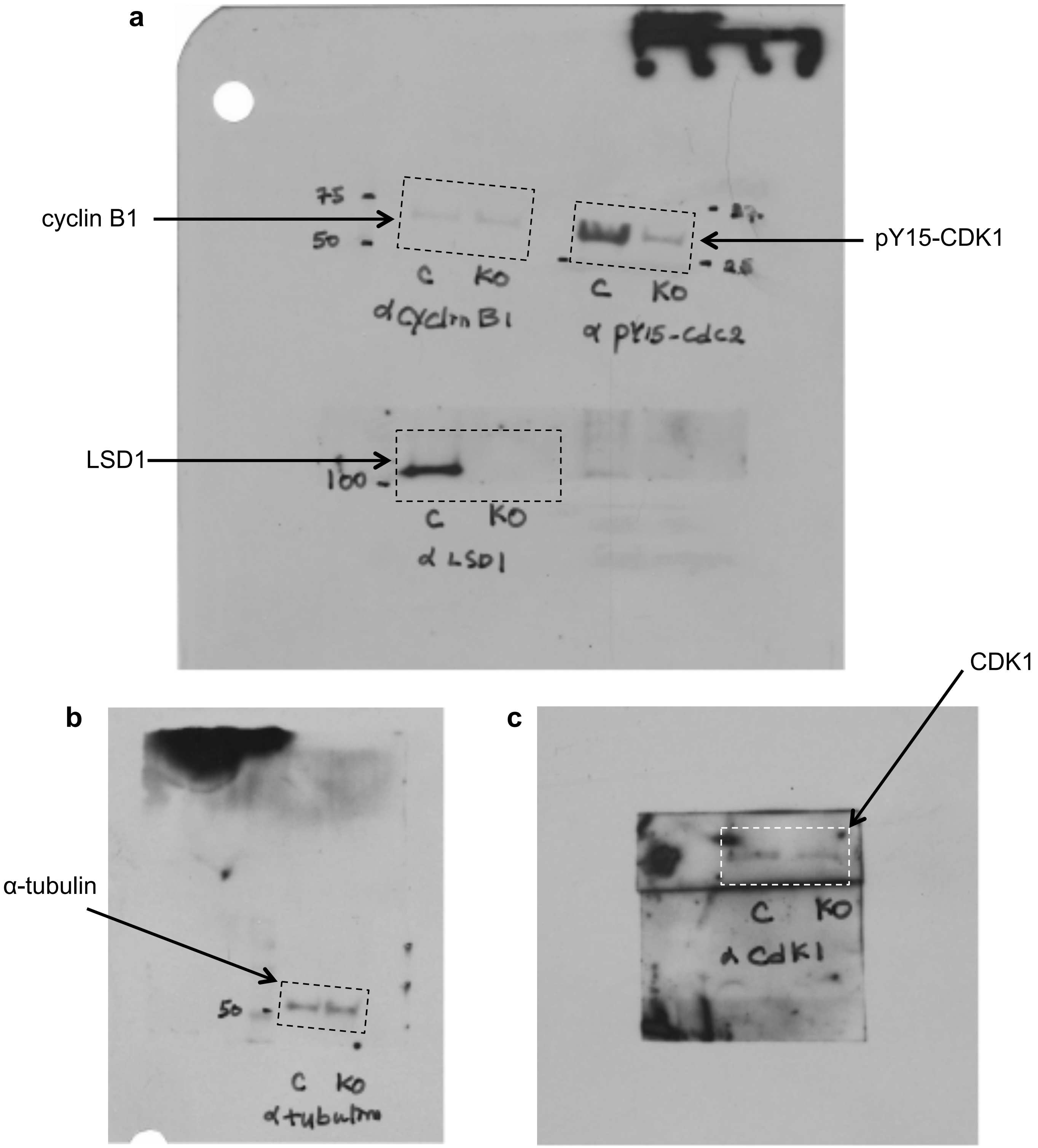

Supplementary Figure 9. Full Western blot gels for Figure 3a
Shown are original gels for cyclin B1, LSD1, pY15-CDK1 (**a**), α-tubulin (**b**), and CDK1 (**c**). The regions shown in Figure 3a are indicated.

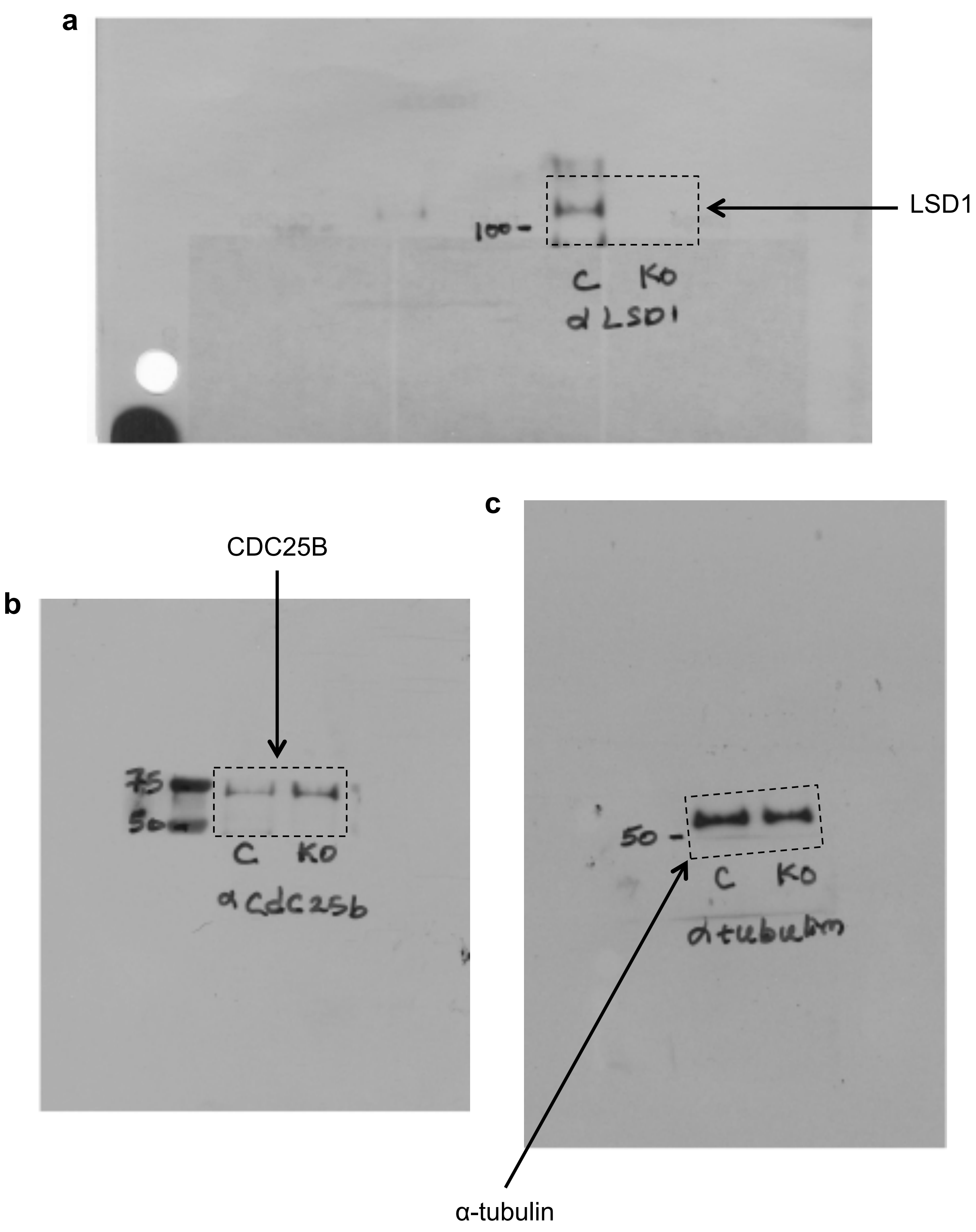

Supplementary Figure 10. Full Western blot gels for Figure 4b
Shown are original gels for LSD1 (**a**), CDC25B (**b**), and α-tubulin (**c**). The regions shown in Figure 4b are indicated.

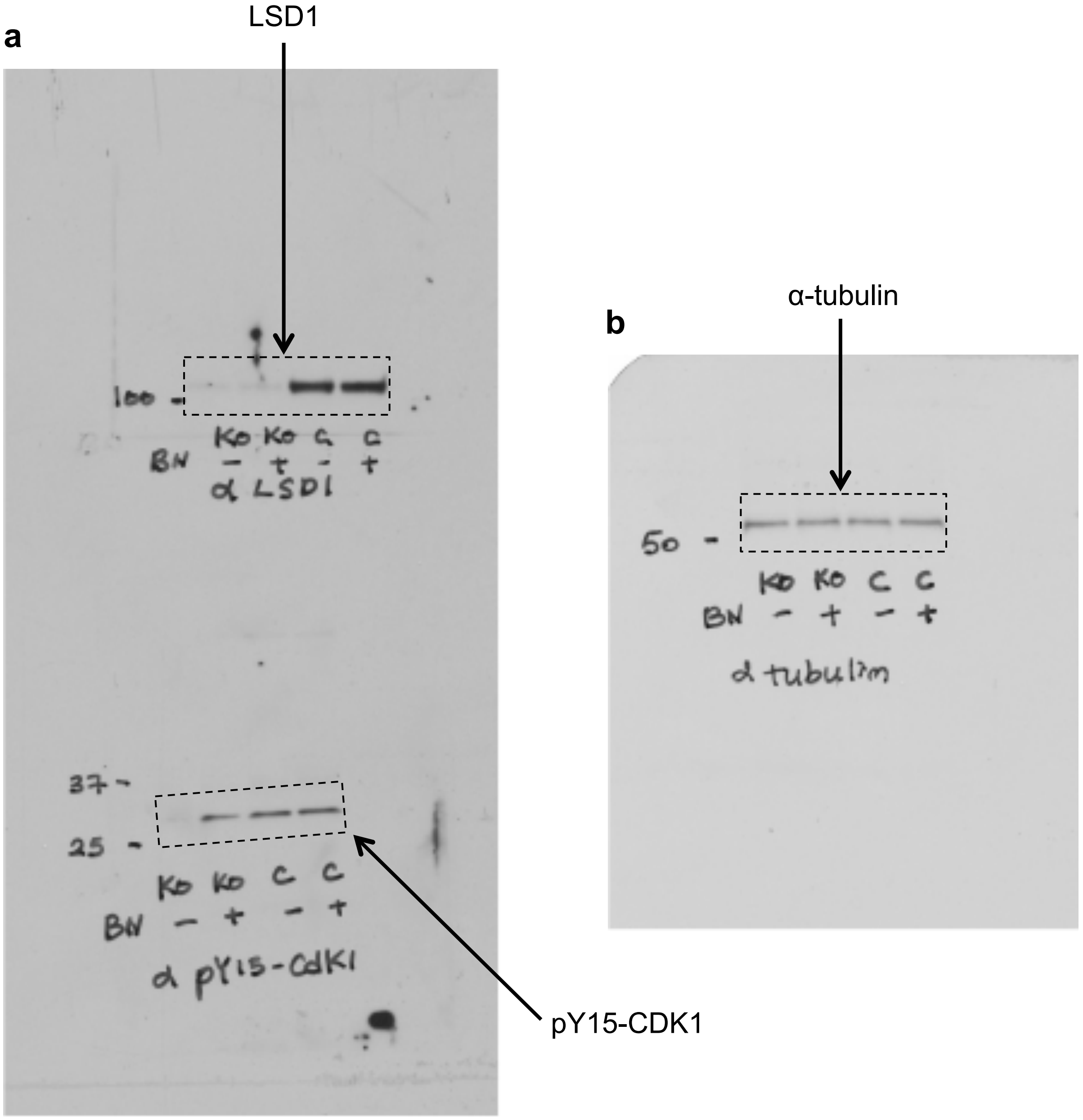

Supplementary Figure 11. Full Western blot gels for Figure 4f
Shown are original gels for LSD1, pY15-CDK1 (**a**) and α-tubulin (**b**). The regions shown in Figure 4f are indicated.

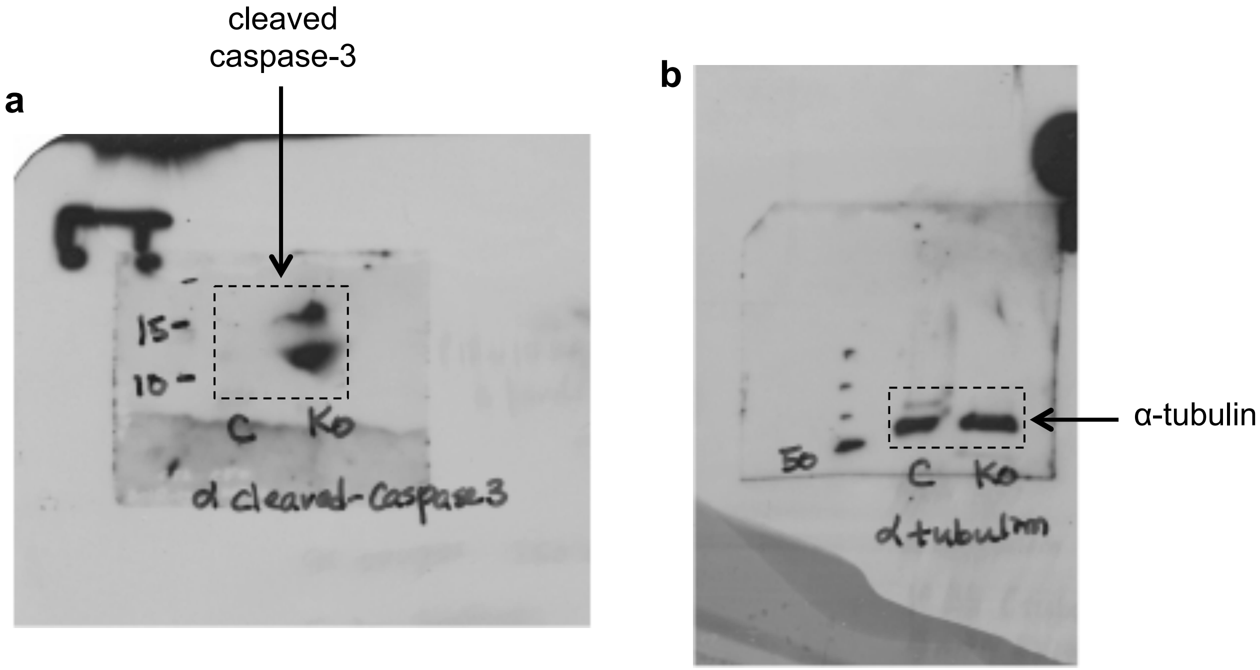

Supplementary Figure 12. Full Western blot gels for Figure 5c
Shown are original gels for cleaved caspase-3 (**a**) and α-tubulin (**b**). The regions shown in Figure 5c are indicated.

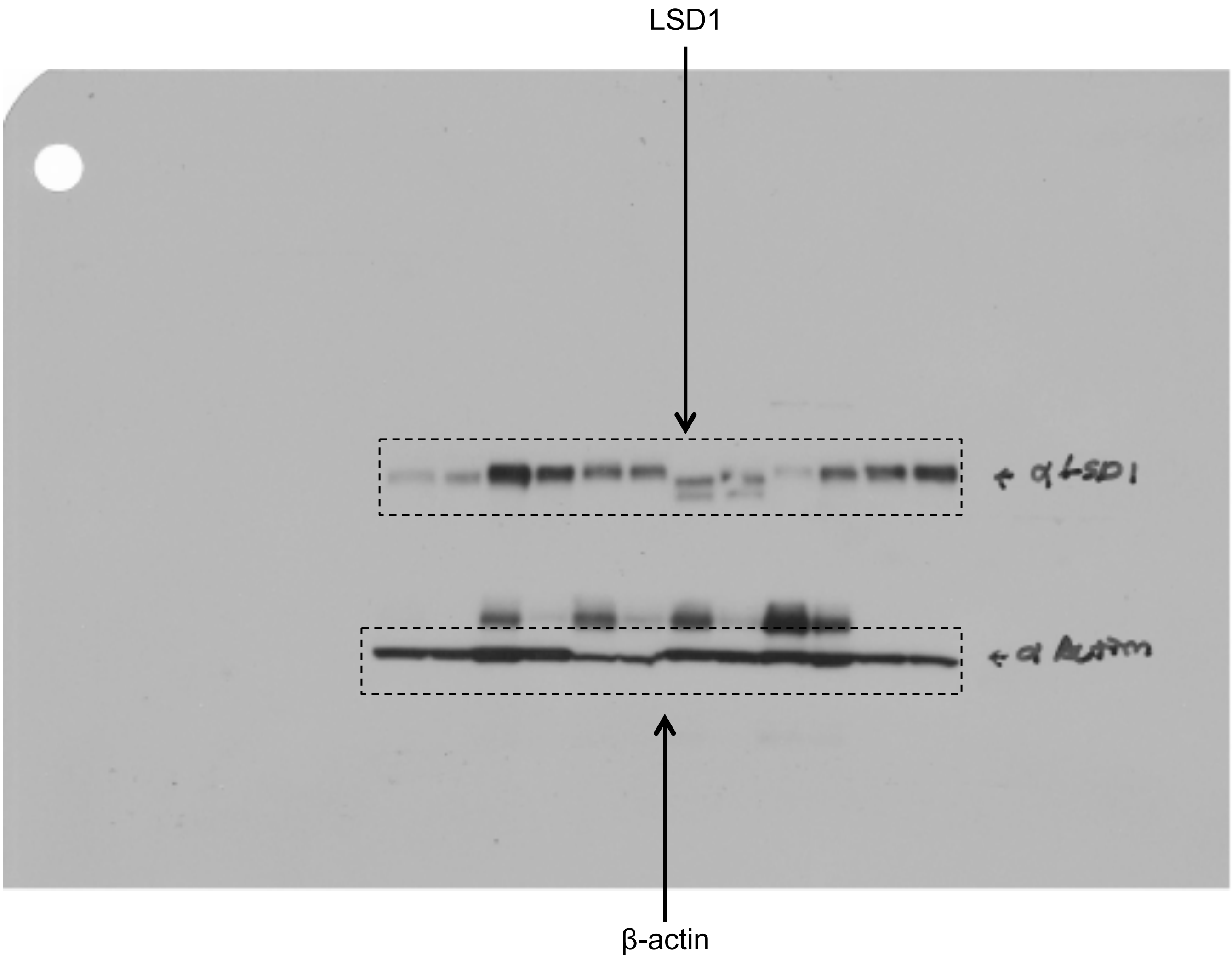

Supplementary Figure 13. Full Western blot gels for Supplementary Figure 1c
Shown are original gels for LSD1 and β-actin. The regions shown in Supplementary Figure 1c are indicated.

Supplementary Table 1. PCR Primers

Primers	Sequences (5' – 3')	Applications
Lsd1 geno-F<br>Lsd1 geno-R	TTGAGTTGGTTGTGAGTCAC<br>AGCGCTAACTTTAGAGCTGG	*Lsd1* genotyping<br>(*WT* 112 bp, *Lsd1*^{fl} 169 bp)
Cre-F<br>Cre-R	GCAAGTTGAATAACCGGAAATGG<br>GCAATTTCGGCTATACGTAACAG	*Cre* genotyping (367 bp)
IAP-F<br>IAP-R	AAGCAGCAATCACCCACTTTGG<br>CAATCATTAGATGTGGCTGCCAAG	*IAP* qRT-PCR
Line1-F<br>Line1-R	GAGACATAACAACAGATCCTGA<br>AACTTTGGTACCTGGTATCTG	*Line-1* qRT-PCR
MLV-F<br>MLV-R	CGAGATCATGGGACAGACCGTAAC<br>CTCCCAGGTGACGATATATGGGAC	*MLV* qRT-PCR
MTA-F<br>MTA-R	ATGTCTTGGGGAGGACTGTGGAAG<br>AGCCCCAGCTAACCAGAACTACAG	*MTA* qRT-PCR
Lsd1-F<br>Lsd1-R	AGCGGGCCAAGGTAGAATACA<br>ATGGGGAAGTCGGCTTTGAAA	*Lsd1* qRT-PCR
Cdc25b-F<br>Cdc25b-R	GAGTGATTTAAAGGATGACGAG<br>ATGATGAGATCCTGTTCCTC	*Cdc25b* qRT-PCR
Wee2-F<br>Wee2-R	AGAGAATTACCAACACCTCC<br>GATCATGTTCTTGAGTAGACC	*Wee2* qRT-PCR
Bcl2-F<br>Bcl2-R	ATGACTGAGTACCTGAACC<br>ATATAGTTCCACAAAGGCATC	*Bcl2* qRT-PCR
Bax-F<br>Bax-R	ATATTGCTGTCCAGTTCATC<br>CCTTTTTGCTACAGGGTTTC	*Bax* qRT-PCR
Bik-F<br>Bik-R	CTCTGAGACTCCCAGCATGA<br>GACACAGGTCCATCTCATCG	*Bik* qRT-PCR

Supplementary Table 2. Antibodies

Antibodies	Vendors; Cat. No.	Dilution	Applications
LSD1	Cell Signaling; 2139	1:200	IHC
LSD1	Cell Signaling; 2139	1:1,000	Western
CDC25B	Sigma; AV41336	1:200	IHC
CDC25B	Sigma; AV41336	1:50	IF
CDC25B	Cell Signaling; 9525	1:1,000	Western
pY15-CDK1	Cell Signaling; 9111	1:1,000	Western
CDK1	Cell Signaling; 9112	1:1,000	Western
cyclin B1	Cell Signaling; 4135	1:1,000	Western
cleaved caspase-3	Cell Signaling; 9661	1:1,000	Western
α-tubulin	Cell Signaling; 2114	1:1,000	Western
α-tubulin	Cell Signaling; 8058	1:100	IF
γ-H2AX	Cell signaling; 7918	1:100	IF
CREST antisera	Antibodies Inc.; 15-235-T	1:100	IF
H3K4me1	Cell signaling; 9723	1:200	IHC
H3K4me2	Cell signaling; 9725	1:500	IHC
H3K9me1	Cell signaling; 14186	1:200	IHC
H3K9me2	Cell signaling; 9753	1:200	IHC
H3K9ac	Abcam; ab32129	1:100	IHC
H3K27ac	Abcam; ab4729	1:500	IHC
H3S10ph	Cell signaling; 9706	1:100	IHC
H3S28ph	Cell signaling; 9713	1:50	IHC
